# Advancements and challenges of onboard carbon capture and storage technologies for the maritime industry: a comprehensive review

**DOI:** 10.1007/s40868-024-00161-w

**Published:** 2025-01-13

**Authors:** Yaseen Adnan Ahmed, Iraklis Lazakis, George Mallouppas

**Affiliations:** 1 Department of Naval Architecture, Ocean and Marine Engineering, University of Strathclyde, Glasgow, G41 4PP UK; 2 CMMI–Cyprus Marine & Maritime Institute, CMMI House-Vasileos Pavlou Square, P.O. Box 40930, 6023 Larnaca, Cyprus

**Keywords:** Onboard carbon capture and storage, Carbon neutral shipping, Maritime emissions challenges, Comparative assessment

## Abstract

In response to the growing demand of reducing greenhouse gas (GHG) emissions within maritime sector, Onboard Carbon Capture and Storage (OCCS) technologies provide as key solutions for tackling carbon dioxide (CO_2_) emissions from ships. This review paper offers a comprehensive overview of recent developments, challenges, and prospects of Carbon Capture and Storage (CCS) technologies considering specifically for onboard ship applications. Various Carbon Capture (CC) methods, ranging from post-combustion and pre-combustion capture to oxy-fuel combustion, are critically analysed concerning their operating principles, advantages, disadvantages and applicability in the maritime context. Temporary onboard CO_2_ storage is examined in its gaseous, supercritical, solid, and liquid states. In this regard, solid and liquid forms are found promising, although solid storage is not yet commercially mature. The review also addresses the challenges in implementing the CC technologies on ships, including space constraints, energy requirements, safety concerns, and economic viability. A comparative assessment is conducted to determine the most promising OCCS technologies. The study finds that post-combustion CC by chemical absorption requires more space than cryogenic and membrane separation, with the latter two deemed viable options, albeit with trade-offs in energy consumption and cost. The study would provide valuable insights and ideas for further research in the field of OCCS technologies.

## Introduction

### Background

It is important to control the significant increase in GHG emissions to address climate change. These gases trap heat in the atmosphere, causing global warming, rising sea levels, and extreme weather events, which harm ecosystems and human health. Reducing emissions helps stabilize the climate and protect the planet's future. In 2019, carbon dioxide (CO_2_) emissions from fossil fuel combustion alone reached 36.7 gigatons (Gt), contributing significantly to total GHG emissions of almost 50 Gt of CO_2_ equivalents (CO_2_,e)—a 40% increase compared to 1990 [1]. Even the brief decline in emissions in 2020 due to the COVID-19 pandemic [2] could not stop the upward trend, underlining the need for effective emission reduction strategies across all sectors.

This environmental crisis is unfolding during rapid global human development and industrial progress since the 20th century. These improvements have raised living standards, but at a significant cost to the environment. Industrial activities have greatly increased the concentration of CO_2_ in the atmosphere, worsening global warming and extreme climate conditions, as shown in recent studies [3, 4]. The energy and transport sectors are the main contributors, producing over two-thirds of GHG emissions [5]. Of particular concern is the transport sector, which accounts for around 25% of global emissions [6], with the shipping industry alone responsible for 12%—almost one billion tonnes annually [6]. The expected increase in global trade, which is expected to increase by almost 40% by 2050 [7], complicates this problem further. As economies grow, so does the demand for maritime transport, leading to projections of future GHG emissions. Forecasts for the year 2050 vary, with the International Maritime Organisation (IMO) predicting a range of 1100–2350 megatonnes per year (Mt/year) for maritime CO_2_ emissions [6]. The World Meteorological Organisation (WMO) also confirms that global warming is deviating from the targets set out in the Paris Agreement [8]. These targets include limiting global warming to well below 2 °C, striving for 1.5 °C, achieving net-zero emissions by 2050, enhancing resilience to climate impacts, and mobilising $100 billion annually to support developing nations in their climate efforts.

Considering the above, a number of measures have been suggested throughout the world to tackle this issue. In this respect, the European Union (EU) and China have set ambitious goals to combat climate change. The EU, along with its member states, is committed to making the European economy carbon-free by 2050 [9]. China has set a target to achieve carbon neutrality by 2060 [10]. Japan is planning to shift to LNG as a bridging fuel and is testing dual-fuel internal combustion engines (hydrogen/ammonia) on small coastal ships, with plans to use them on larger ocean-going ships once the technology advances [11]. Norway is targeting a 45% reduction in carbon emissions from domestic shipping before 2030, employing legislative measures and financial incentives to promote low-carbon initiatives [12].

In the UK, Lloyd’s Register has analysed the factors affecting the construction and operation of zero-emission ships, highlighting the major challenge of high operating costs when converting ships [13]. In addition, in 2018, the IMO adopted an initial strategy to reduce GHG emissions from ships, which sets out specific targets and phased measures to reduce emissions [14]. In June 2021, the IMO adopted important short-term measures with the aim of reducing the carbon intensity of all ships by at least 40% by 2030 [7]. More recently, in July 2023, the IMO adopted a revised GHG strategy that significantly raises the ambition for the global shipping industry. In contrast to the original target of a 50% GHG reduction by 2050, the updated strategy sets stricter targets [15]. Starting from 2008, the new targets include a 20% reduction in waking GHG emissions by 2030, a 70% reduction by 2040 and a commitment to achieve net-zero emissions by or around 2050 [15].

However, the future of the shipping industry depends on global standards being set and new technologies being deployed. There is an urgent need for action as the maritime sector plays a leading role in tackling this environmental challenge. This situation requires creative solutions, strict regulations and international cooperation to ensure a more sustainable future.

### Alternative decarbonisation solutions for maritime transportation

To meet the IMO’s stringent targets [15], the shipping industry needs to adopt a new operational paradigm where innovative materials, technologies, processes, designs and practises are rapidly introduced for both new and existing ships. Decarbonisation strategies suitable for ships can be broadly divided into the following five key categories [16]:

#### Logistics and digitalisation

Strategies such as slow steaming, weather routing, route optimisation and the integration of ship energy management systems are essential to achieving emissions reduction targets.

#### Hydrodynamics

Innovations in hull hydrodynamics, hull coating and air lubrication can have a significant impact on the energy efficiency of ships.

#### Machinery

Improving engine efficiency, optimising propulsion systems, using devices to increase propulsion efficiency and implementing waste heat recovery are crucial to improving the energy efficiency of ships.

#### Alternative energy

The maritime sector is exploring alternative fuels such as ammonia, hydrogen, methanol, liquefied petroleum gas (LPG), biofuels (such as bio-oils and hydrogen-treated vegetable oils) and liquefied natural gas (LNG), which is mainly used in LNG carriers. In addition, fuel cells, hybrid systems and wind and solar assist technologies could reduce emissions and improve energy efficiency. These alternatives promise to reduce GHG emissions, even if their widespread introduction is associated with challenges such as engine compatibility and bunker infrastructure.

#### After treatment

As zero-emission technologies evolve, CCS can serve as a medium- to long-term interim solution to reduce CO_2_ emissions while reducing competition for carbon-neutral fuels.

**Fig. 1:**
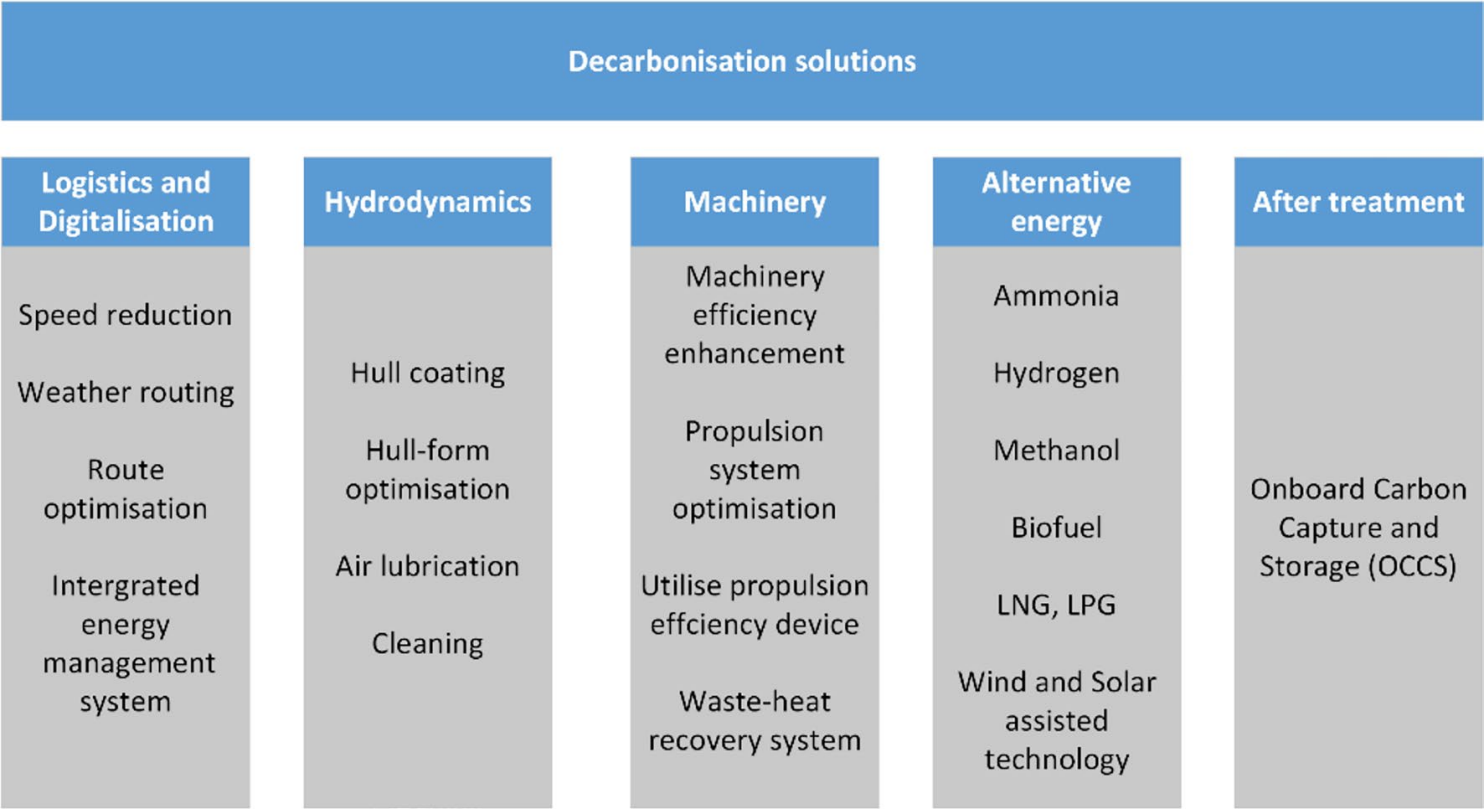
Solutions that can contribute to decarbonise shipping

Figure 1 shows the solutions for decarbonisation, which are divided into five alternatives. Numerous studies have analysed the effectiveness of these measures and strategies in improving the energy efficiency of ships and reducing GHG emissions. While biofuels are promising due to their environmental friendliness, energy density and fungibility and have the potential to reduce GHG emissions by 100% based on a well-to-wake analysis, practical challenges such as storage, engine compatibility and bunker infrastructure limitations limit their applicability [17] and [18]. The feasibility of utilising biofuels depends on the type of feedstock used, with newer generations of biofuels offering potential solutions to some of these challenges. Furthermore, tackling emissions in shipping is primarily about improving the efficiency of main and auxiliary engines [19]. Waste heat recovery with systems such as the organic Rankine cycle is very promising for shipping [20–22]. Scientists have also looked at combined cooling, heating and power (CCHP) cycles fuelled by waste heat to meet various onboard energy needs [23].

On the other hand, the majority of individual approaches relevant to logistics and digitalisation, with the exception of slow steaming, generally only lead to a limited reduction in GHG emissions, as extensive studies such as the comprehensive study by Balcombe et al. [24] showed. Their findings suggest that technologies like route optimisation and fuel management offer modest benefits, but alone they are insufficient for significant decarbonization. Slow steaming, however, provides more substantial reductions. The study emphasizes that achieving the target of a 50% GHG reduction by 2050 requires a combination of strategies, including alternative fuels, efficiency measures, and strong policy support.

Optimising the propulsion and energy systems of ships is of central importance for improving energy efficiency. While solar and wind energy technologies are relatively mature, the low power density and volatility of these sources suggest that fuel cell and hybrid technologies will become the dominant energy sources for environmentally friendly ships [25].

Conventional marine fuels such as Marine Diesel Oil (MDO) and Heavy Fuel Oil (HFO) contain high carbon content [26], making the transition to low or zero carbon fuels an urgent concern. In this context, bridge fuels such as LNG [27] and alternative fuels such as hydrogen [28] and ammonia [29] are gaining importance as clean energy options. Orders for new ships indicate a shift towards alternative fuels, with companies such as A.P. Moller-Maersk ordering dual-fuel methanol container ships [30], followed by other industry leaders such as CMA and CGM [31], Cosco [32] and Cargill [33]. In addition, according to Clarksons Research, there were 90 newbuilding orders for ammonia-capable ships in 2022 as a whole, representing 11% of tonnage, while 43 orders (7%) were for methanol-capable ships and 3 for hydrogen-capable ships [34].

In the face of uncertainty about the availability of low-emission fuels, shipowners are adapting their strategies by either upgrading existing ships or building new fleets that can run on both conventional and alternative fuels. This approach recognises that it remains difficult to completely eliminate emissions from ships unless a complete reliance on alternative fuels becomes feasible. In this regard, one possible solution is the capture of carbon emissions from ships using commercially recognised CCS technologies. As OCCS would utilise a proven technology, it requires less research and development compared to alternative fuels. In addition, OCCS can achieve significantly higher emission reduction rates than the fuel-saving measures mentioned above and accelerate progress towards the IMO target of 85% emission reduction per ship [13]. However, the amount of energy that OCCS requires at the expense of fuel must be taken into account.

### Current development of OCCS

This subsection provides an overview of key CCS projects in the maritime sector, focusing on the CC-Ocean [35], EverLoNG [36], decarbonICE [37], Green Marine [38], and emerging developments under the Bulk Carrier Carbon Capture [39] and REMARCCABLE [40] projects. Although available information from open sources is limited, the primary objectives and progress of these initiatives are presented below.

**Fig. 2:**
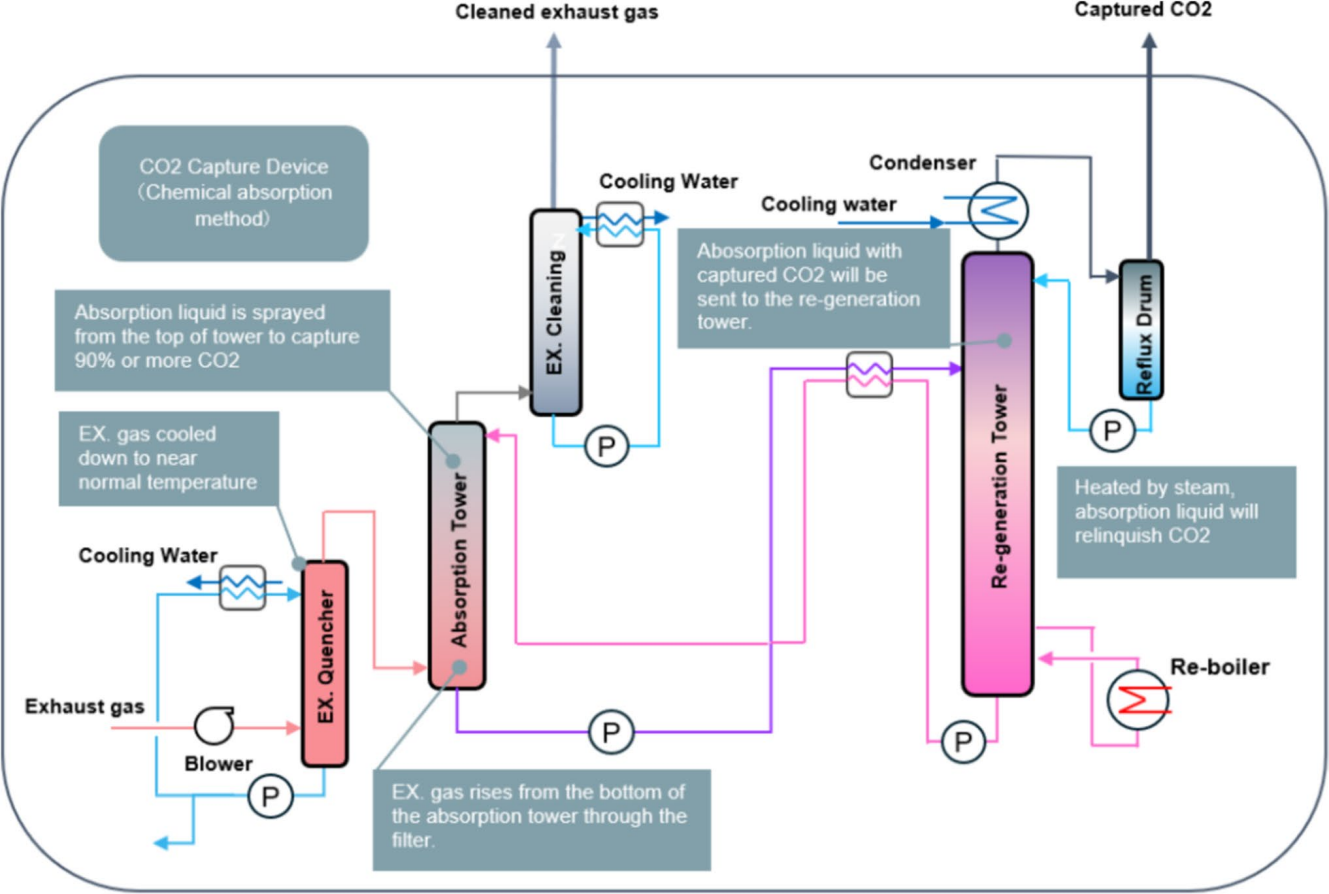
Schematic diagram of the CCS technologies for CC-Ocean according to [35]

The CC-Ocean project [35] is a groundbreaking initiative focused on validating onboard CO_2_ capture systems aboard the Corona Utility, an 88,000-tonne bulk carrier. Led by Mitsubishi Shipbuilding Co. Ltd. and Kawasaki Kisen Kaisha Ltd., and supported by Japan’s Ministry of Land, Infrastructure, Transport, and Tourism, this project employs post-combustion chemical absorption to capture CO_2_ from the exhaust gases of marine engines. Six months of operation showed that the system met the initial project targets in terms of CO_2_ quantity, ratio, and purity (greater than 99.9%), proving the feasibility of CO_2_ capture in a commercial maritime context [41]. To understand the schematic of OCCS arrangement of CC-Ocean, Fig. 2 is referred.

In the EverLoNG project [36], TotalEnergies and Carbotreat are advancing Ship-Based CC (SBCC) technology by installing a CO_2_ capture prototype aboard an LNG-powered carrier. This system aims to capture ten tonnes of CO_2_ over 3000 h of operation, with additional testing planned on other vessels. The project aims to demonstrate a 70% reduction in CO_2_ emissions from ships, furthering the development of market-ready SBCC solutions.

The decarbonICE project [37] is focused on an innovative cryogenic CCS system, capturing CO_2_ from exhaust gases, converting it into dry ice, and storing it in seafloor sediments. This project, initiated in 2019, is exploring low-energy CO_2_ capture technologies (predicted energy penalty below 10%), with the goal of achieving carbon-negative shipping when integrated with future carbon–neutral fuels.

Green Marine [38], funded by Horizon Europe, aims to accelerate climate neutrality in waterborne transport by retrofitting existing ships with emission control technologies. The project includes developing protocols for retrofitting engines and installing systems for CO_2_ capture and energy saving, with the MV Coruisk, a passenger ferry in Scotland, set to serve as the test vessel for these technologies.

Under development, two key projects are advancing CC systems for ships. The Bulk Carrier Carbon Capture project Marine [39], approved in principle by Bureau Veritas (BV), involves two bulk carriers, the Tianjin Venture and CSSC Wan Mei, equipped with a CO_2_ capture system that uses an organic amine solution for chemical absorption. Laboratory tests have demonstrated a CO_2_ capture rate of over 85%.

The REMARCCABLE project [40], approved by the American Bureau of Shipping (ABS), is testing a CO_2_ capture system aboard a medium-range tanker operated by Stena Bulk. This project will evaluate the performance of the system over a two-year period, with sea trials expected to involve CO_2_ capture during deep-sea voyages. Stena Bulk intends to extend the use of this system beyond the pilot phase, indicating strong potential for long-term integration of CCS technology in maritime operations.

Based on the limited information provided by the aforementioned OCCS projects, it is clear that the amount of CO_2_ that needs to be captured and stored during a typical voyage depends on several factors, such as the size of the vessel, the type of fuel used, and the operational conditions. While precise figures on CO_2_ mass and storage volumes for specific ships are not readily available, these ongoing projects have provided target capture rates and system designs. For instance, the CC-Ocean project [35] and EverLoNG project [36] focus on achieving CO_2_ capture rates of around 70–85% during operation. These systems are designed to capture and store CO_2_ from exhaust gases emitted by marine engines, but the precise volume of CO_2_ captured per day will vary depending on the engine load and fuel type used during the voyage. For example, in the EverLoNG project [36], the objective is to capture up to 10 tonnes of CO₂ over 3000 operational hours, giving an idea of the scale of CO₂ capture required.

In terms of storage, the mass and volume required depend on the method of CO_2_ storage, whether it’s stored as liquid CO_2_ or solidified into dry ice (as in the decarbonICE project [37]). The space required for onboard CO_2_ storage is also influenced by the storage method, with liquid CO_2_ requiring significant tank volumes, while solid CO_2_ in the form of dry ice would require more specialised storage systems. The storage capacity of a typical vessel, such as a bulk carrier or tanker, would need to be tailored to the specific CO_2_ capture and storage systems installed. The required space for different technologies is further discussed in Sect. 5, and the various factors influencing storage volume and mass are covered in Sect. 7.2.2.

The above is also related to the space required for CO_2_ purification as it depends on the purification method, the scale of CO_2_ capture, and the design of the system. For compact technologies like membrane separation, the space needed could be as little as a few cubic meters, while more complex systems such as cryogenic separation may require larger spaces, potentially hundreds of cubic meters due to the need for cryogenic storage tanks. Chemical absorption and oxy-fuel combustion systems also demand significant space for towers, reactors, and integration with the ship's infrastructure. Additionally, the amount of CO_2_ captured and stored plays a significant role in determining the space requirement, with larger ships needing more space for purification and storage systems. So far, the values are not specific and vary case to case.5

On the other hand, the rate of CO_2_ capture while cruising varies by technology and ship type. For example, the Bulk Carrier Carbon Capture project [39] targets a CO_2_ capture rate exceeding 85% from exhaust gases, while the REMARCCABLE project [40] aims for continuous CO_2_ capture during deep-sea voyages. The specific rate of CO_2_ capture is detailed in Sect. 7.2.4, which covers different technologies and their respective efficiencies.

### Aim of this study

CCS technologies are primarily used for onshore projects, such as Shell Canada's Quest in Alberta, where the CCS facility captures over one million tonnes of CO_2_ annually from the Scotford Upgrader and has stored over 7 million tonnes since 2015 [42], with only a limited number used on ships. The lack of current commercial shipping applications of CCS emphasises the need for further research and development. While substantial progress has been made in onshore CCS technologies, the application of these systems onboard ships presents unique challenges, such as limited space, high energy requirements, and the need for cost-effective solutions. These challenges highlight the urgency for dedicated research to adapt and develop CCS technologies suitable for maritime use.

The main objective of this study is to conduct a comprehensive review of relevant articles and project reports focussing on Onboard Carbon Capture and Storage (OCCS) to understand the operating principles, advantages and disadvantages, and recent advances of CCS technologies for onboard applications. This review aims to fill the gap in the existing literature, which predominantly focuses on land-based CCS applications. By analysing a wide range of sources, this paper provides a deeper understanding of how CCS technologies can be adapted to the unique conditions of ships. This paper also addresses the identification of OCCS-specific challenges through a thorough and structured literature review, followed by a comparative assessment of different CC technologies to identify promising solutions based on their potential to address the identified challenges. Temporary on-board CO_2_ storage options, including gaseous, supercritical, solid and liquid forms, are also investigated as an integral part of the analysis.

The findings of this overview study will assist stakeholders in identifying the key challenges hindering the implementation of commercially viable onboard CCS technologies. In addition, the study provides insights for selecting the most promising technology based on preferences in terms of space, energy requirements or cost, while taking other challenges into account. This dual approach of reviewing existing technologies and assessing the specific needs of the maritime industry offers a more comprehensive analysis and a valuable framework for future developments.

The paper is organised as follows: Sect. 2 outlines the methodology for selecting relevant literature and conducting a comparative assessment. Following this, Sect. 3 explores the potential of OCCS through literature and critical review. In Sect. 4, an overview of different CC technologies, is presented including their working principles, advantages, disadvantages, and different research outcomes for onboard applications. Section 5 delves into the scope of onboard temporary CO_2_ storage in the form of gaseous, supercritical, solid or liquid state. Section 6 lists the challenges identified for OCCS implementation. Subsequently, Sect. 7 conducts a comparative assessment among different CC technologies, considering their potential to be installed onboard, while addressing the challenges mentioned in Sect. 6. Finally, Sect. 8 concludes with the findings of this review paper.

## Methodological approach

The methodology applied in this study follows a systematic approach to gather knowledge about the OCCS technologies and the associated challenges for on-board implementation and to conduct a comparative study to determine the potential of each technology to overcome these challenges. The methodology comprises five main steps: (a) literature search and selection, (b) data extraction, (c) data synthesis, (d) data analysis and (d) comparative assessments and discussion.

**Fig. 3:**
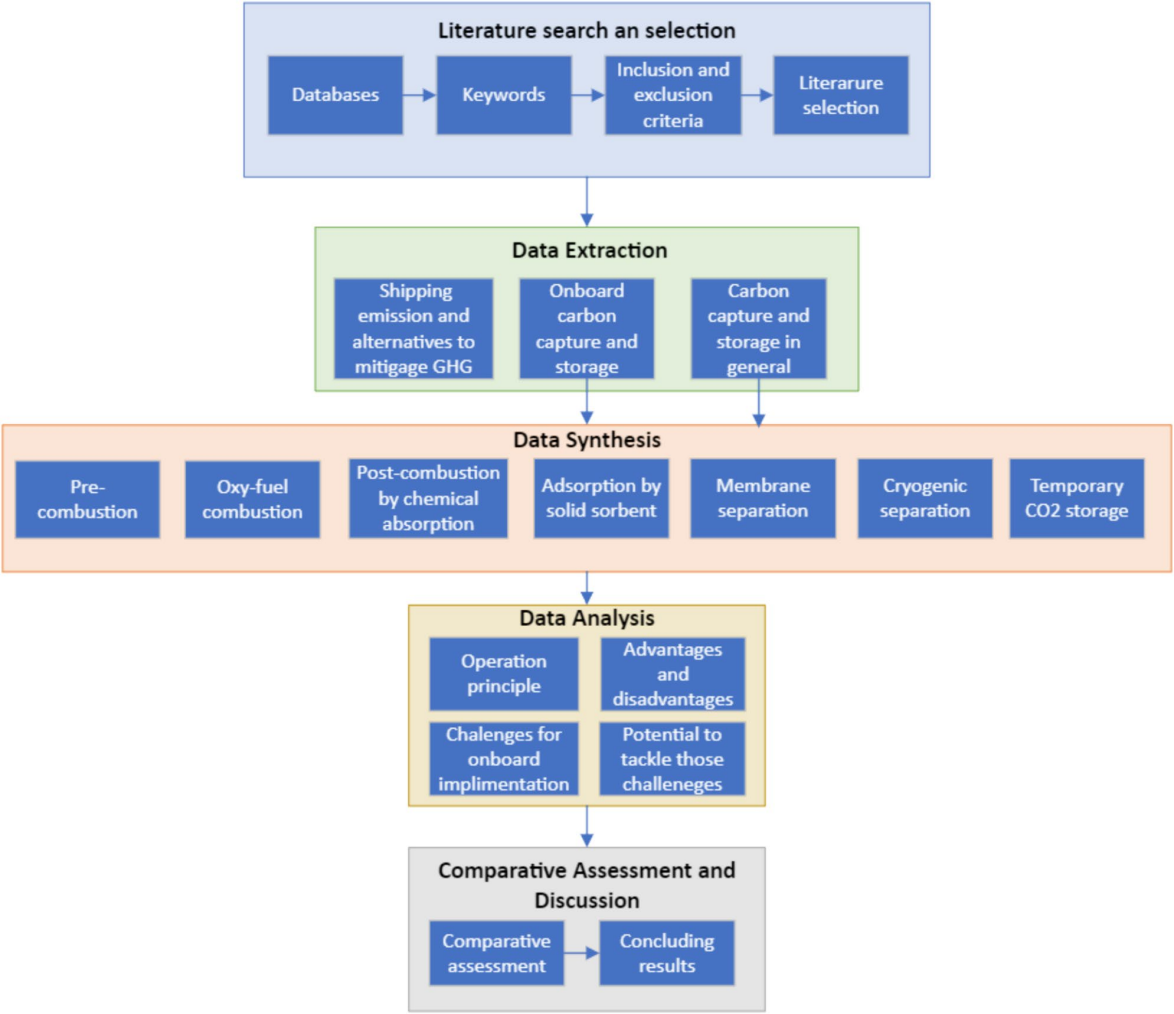
Methodological flow chat

Figure 3 illustrates the methodological approach of this study and provides a detailed insight into the components of the individual steps. These steps are explained in more detail in the following paragraphs of this section.

### Literature search and selection

A comprehensive search was conducted in several databases, including ScienceDirect, Springer, MDPI, IEEE Xplore, ResearchGate, ACS publications, SAGE journals, Frontiers, Taylor & Francis, SSRN, institutional libraries and official websites. The aim was to find literature on Onboard Carbon Capture and Storage (OCCS) for the period 2013 to 2023 using keywords such as 'Onboard/Shipboard Carbon Capture and Storage', ' Carbon Capture and Storage for ships' and 'Carbon emission reduction technologies for ships' The search yielded 46 relevant publications dealing specifically with OCCS.

Although there is an abundance of publications dealing with CCS for industrial applications, there are few that deal with on-board implementation. To fill this gap and comprehensively analyse the operating principles, advantages, disadvantages and recent developments of CCS for both industrial and on-board applications, the authors applied the snowball method. The snowball method consisted of using source lists in relevant articles to create a network of related literature. This method was particularly useful given the abundance of literature on industrial CCS and allowed the authors to selectively choose 52 supporting documents. This curated selection served to justify and enhance the key information presented in the study, resulting in a well-rounded examination of both general CCS principles and specific considerations for on-board implementation. Table 1 contains the keywords used, the inclusion and exclusion criteria and the overall selection of 46 and 52 reports/papers for OCCS and CCS in general, respectively.

**Table 1:** Literature selection for OCCS and CCS in general

Database	Keywords	Inclusion criteria	Exclusion criteria	Final selection (OCCS)	Final selection (general CCS)
ScienceDirect	‘Onboard/Shipboard CC and Storage’, ‘CC and Storage for ships’, ‘carbon emission reduction technologies for ships’	OCCS/CCS methods, recent advancement, implementation challenges for ships, overcoming strategies, comparison assessments, explains advantages and disadvantages	Outdated studies, irrelevant topics, OCCS/CCS supply chain, ship/CO_2_ transportation, CO_2_ sequestration	17	35
Springer				1	3
MDPI				2	1
IEEE Xplore				3	–
ACS publications				2	1
SAGE journals				1	–
Wiley				–	2
Frontiers				1	1
Chemistry Europe					1
Taylor & Francis				2	–
SSRN				1	–
Institutional libraries				3	1
Information/reports from Official webpages				13	7

Furthermore, literature on shipping emissions statistics, chemical priorities of CO_2_, different codes, alternative initiatives for GHG reduction, achieving IMO's 2050 emission target, and Technology Readiness Levels (TRL) for emission reduction technologies were considered. In total, 37 reports/papers of relevant literature were identified for this purpose.

### Data extraction

**Fig. 4:**
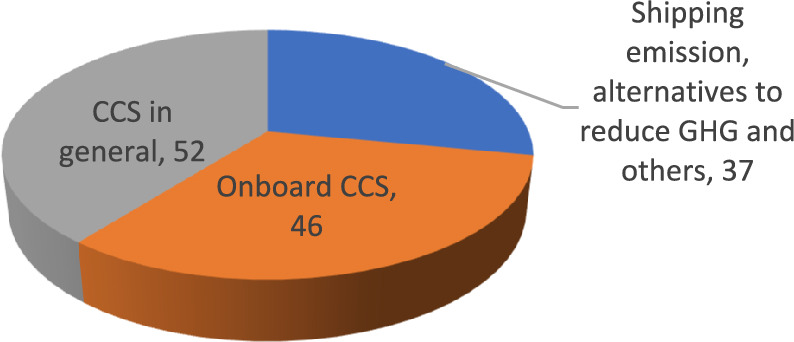
Data extraction based on respective criteria

The literature selection section clarifies that the authors employed the keywords and databases to pinpoint relevant reports/papers related to OCCS, and snowballing technique for CCS in general. This approach facilitated the extraction of data into three distinct groups, streamlining the subsequent investigation in this study. Figure 4 illustrates the distribution of identified reports/papers based on their respective criteria, which are onboard CCS, CCS in general and other papers/reports explain shipping emission, alternatives to reduce GHG and so on.

### Data synthesis

**Fig. 5:**
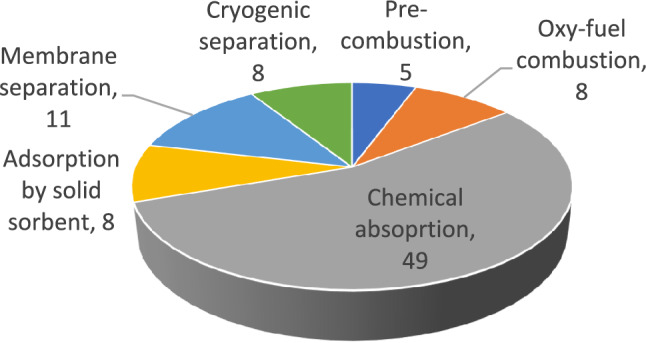
Data organised based on different CC technologies

**Fig. 6:**
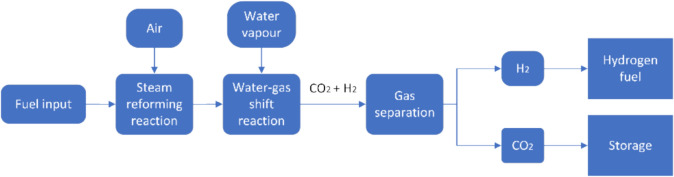
Schematic of pre-combustion capture

The reports/papers discussing the OCCS and CCS in general were re-organised focusing on various onboard CC and storage technologies. This included the examination of pre-combustion, oxy-fuel combustion, post-combustion by chemical absorption, adsorption by solid solvents, membrane separation, and cryogenic separation. Additionally, the temporary storage of CO_2_ in gaseous, subcritical solid and liquid forms was considered as an integrated component of CC. Figure 6 illustrates the distribution of identified reports/papers for different CC technologies. It is important to note that pertinent reports/papers discussing multiple CC technologies, such as review papers or those conducting comparative assessments, are counted as inputs for each respective CC technology when preparing Fig. 5.

### Data analysis

The study included a thematic analysis of reports/papers for each CC technology, focussing on the operating principles, advantages, disadvantages, challenges for on-board installation and their possible solutions. In addition, the thematic analysis not only highlighted the challenges but also looked for possible solutions. This approach contributed to a more holistic assessment of the individual CC technologies and provided insights not only into the hurdles, but also into the possible ways to overcome them in the context of on-board applications. By categorising the reports/papers thematically, the study aims to provide a differentiated understanding of the potential applications of the individual CC technologies in the maritime industry.

### Comparative assessment and discussion

A comparative assessment was then carried out based on the findings from the thematic analysis of the reports/papers. As there are hardly any reports/papers dealing specifically with OCCS, the authors considered more general CCS-based reports/papers to gain additional information and improve the study. The assessment aimed to address the challenges identified in the thematic analysis of reports/papers in the implementation of onboard CC technologies. The assessment was conducted in two different phases. In the first phase, the focus was on determining the feasibility of the technologies for on-board installation. A follow-up assessment was then carried out, focussing on the remaining challenges that the CC technologies need to overcome.

The final conclusions were based on the suitability of on-board CC technologies, considering both the feasibility of installation and the capabilities of the technologies to overcome the identified challenges.

## Potential of OCCS

In the quest to decarbonise the maritime industry, OCCS could be a promising solution for reducing CO_2_ emissions from ships. Although research in this field is relatively scarce, recent studies have triggered a wave of innovation and exploration, paving the way for a sustainable future on the high seas.

Lloyd's Register's (LR) readiness assessment on OCCS [43] explores alternative solutions for the capture and storage of CO_2_ emissions on ships. Two main methods are described: pre-combustion capture, where the ship's fuel is converted into a gas and the CO_2_ is captured before combustion, and post-combustion capture, which includes chemical absorption, membrane technology, cryogenic capture, oxy-fuel combustion and capture with solid sorbents. The captured CO_2_ can be stored on-board in liquid or solid form but must be offloaded for further processing in a harbour, either for permanent underground storage or for conversion into materials for various industries.

Luo and Wang [44] laid the foundation for maritime CC by investigating solvent-based processes for capturing CO_2_ from the energy system of a cargo ship. Their pioneering work opened new avenues and piqued the interest of researchers and industry experts. Building on this initial study, Feenstra et al. [45] took a significant leap forward by investigating the feasibility of integrating post-combustion CO_2_ capture technologies specifically for maritime applications. Various solvents, including a 30% wt aqueous solution of mono-ethanolamine (MEA) and 30% wt aqueous piperazine (PZ), were examined in detail. In addition, the potential of ammonia (NH_3_) as a solvent for CC on-board liquefied natural gas (LNG)-fuelled CO_2_ tankers has been investigated, highlighting the versatility of this approach [46].

One of the most intriguing facets of OCCS lies in its ability to liquefy the captured CO_2_ for storage. This transformative step is the answer to the challenge of effectively dealing with captured emissions. In addition, there are futuristic visions that propose capturing CO_2_ from ship exhaust, which could then be subjected to cryogenic processes and converted into dry ice, offering a new perspective on CC and utilisation [47]. While the method temporarily captures CO_2_ and delays its release, it requires a closed CO_2_ capture loop to be effective. However, the feasibility of this method remains uncertain due to its low conversion efficiency. The maritime industry envisions a hydrogen-based future in which containerised liquefied CO_2_ becomes a valuable feedstock to produce synthetic carbon fuels. This vision depends on the large-scale production of hydrogen (H_2_) from renewable sources such as solar or wind energy. When H_2_ is abundant, scientists and process/chemical engineers can synthesise various synthetic fuels, including methane (CH_4_) or methanol (CH_3_OH), from H_2_ and CO_2_. This transformative process not only reduces carbon emissions, but also offers the opportunity to create sustainable energy sources and lead the industry towards a greener horizon [48].

In the search for more efficient and sustainable CC technologies, researchers have proposed several innovative solutions. These advances are essential to overcome the limitations of conventional land-based CC processes. Offshore environmental conditions, such as high salinity, humidity, and harsh marine elements, present unique challenges for the durability and performance of CC technologies. One key difference between offshore and land-based applications is the need for all equipment and materials to be marine-grade or marine-approved. These materials are specifically designed to resist corrosion from seawater and other environmental factors, ensuring long-term reliability. While this requirement increases equipment costs due to the need for specialised materials and certifications, it is essential for ensuring safety and regulatory compliance.

For absorption-based technologies like MEA-based CO_2_ capture systems, a key challenge is the need for water to replenish the MEA solution. Using desalinated seawater offshore could be a practical solution, as it would reduce the need for separate distilled water storage. However, desalinated seawater may contain impurities that could affect the performance and longevity of the MEA solution, requiring extra treatment. If desalinated seawater isn't viable, a separate distilled water tank would be needed, adding complexity to the system. Other limitations include low vapour loading capacity, significant energy consumption during solvent regeneration, large equipment dimensions, high equipment corrosion rates and solvent degradation. The use of advanced solvents [49] and [50], optimised operating conditions [51] and state-of-the-art column internals [52] increases the efficiency of the deposition process. In addition, technologies such as intercoolers [53], reheaters [54] and flue gas precoolers [55] help to reduce energy consumption and make on-board CC a more viable option for large-scale implementation.

Flue gas compression and expansion [56] and the introduction of multiple feeds and semi-clean solvent configurations [57] optimise the capture process and ensure a higher CO_2_ removal rate. Rich solvent recycling systems [58] and square columns [59] further improve the overall efficiency of onboard CC systems, making them more environmentally friendly and economically viable. These advances emphasise the industry's commitment to cleaner, safer and more energy-efficient solutions for CC at sea.

In the area of process integration, innovative technologies such as heat pumps [51] and self-heat recovery techniques [52] offer a holistic approach to improving the overall energy efficiency of onboard CC systems. These integrative methods not only reduce operating costs, but also help to minimise the environmental footprint of CC processes. In addition, process intensification techniques, including reactive absorption [60] and rotating fixed beds [61], have shown remarkable potential. These methods pave the way for smaller, cleaner and safer technologies that make CC an increasingly attractive option for the maritime sector.

In addition, research efforts have focussed on evaluating on-board CC from an Energy Efficiency Design Index (EEDI) perspective. Studies by Stec et al. [26] and Lee et al. [62] have shown that OCCS technology can significantly reduce the EEDI and thus contribute to improved energy efficiency of ships.

In short, ship-based CC is at the forefront of the maritime industry's transition to sustainability. The industry is paying increasing attention to OCCS technology, with initiatives such as the OGCI and Stena Bulk report [63] emphasising its technical feasibility. Pilot implementations such as the small onboard CC system installed on a Japanese coal carrier owned by shipping company K Line [64] demonstrate the practical progress being made in the introduction of OCCS. Although challenges related to high capital expenditure (CAPEX) and operating expenditure (OPEX) remain, OCCS stands out as a proactive and pragmatic solution for significant emission reduction in the maritime sector. As OCCS research and development continues, the technology is poised to mature rapidly, making it a viable and efficient tool on the maritime industry's path to decarbonisation.

## Overview of CC technologies

This section presents CC technologies (CCS) and provides insights into the current status, progress, existing literature and research, with a particular focus on the application of onboard CC. In general, three methods for CC can be distinguished: pre-combustion capture, oxy-fuel combustion capture, and post-combustion capture.

### Pre-combustion capture

#### Operating principle

Pre-combustion capture focuses on the removal of CO_2_ prior to the combustion of fossil fuels. This method includes gasification and reforming processes of fossil fuels with air and water vapour, producing CO_2_ and H_2_. These processes include steam reforming, which produces CO and H_2_, and the water–gas shift reaction, which converts CO to CO_2_, as described by Wang et al. [65]. The resulting CO_2_ and H_2_ are separated using gas separation techniques, with the CO_2_ captured for storage and H_2_ available as fuel for hydrogen gas turbines. Pre-combustion capture is characterised by low-energy consumption and high separation efficiency, allowing almost 90% of CO_2_ to be captured from the fuel source [66]. Figure 6 illustrates the entire pre-combustion capture process flow.

#### Advantages and disadvantages of pre-combustion capture for onboard application

While existing reports/papers examine the advantages and disadvantages of pre-combustion capture for industrial use, this study aims to assess the pros and cons of applying pre-combustion capture technology in the context of its onboard application. Table 2 is prepared in this regard. The information provided in Table 2 is utilised in the comparative study conducted in Sect. 7 of this study.

**Table 2:** Advantages and disadvantages of onboard pre-combustion capture

Advantages	Disadvantages
Production of hydrogen (H_2_) for energy generation [67]	Elevated capital expenditure (CAPEX) for syngas generation components, reactor tanks, and hydrogen fuel engine modifications
Accelerating adoption of H_2_ as an alternative fuel [68]	Less economically favourable compared to post-combustion CC due to higher CAPEX [68]
Reduced energy requirements for CO_2_ compression and storage due to capturing CO_2_ at elevated pressure [68]	Filtration of non-convertible impurities required
The energy demand for the capture and stripping processes is lower compared to post-combustion CC [69]	Disruption in syngas production halts H_2_ production, leading to loss of propulsion and auxiliary generator fuel without a bypass [68]
Higher CO_2_ content in syngas enhances CC efficiency [69]	Need to minimise H_2_ production and storage in advance due to explosive nature, increasing risk mitigation efforts
	Cannot avoid impact on ship stability even though having smaller equipment size compared to post-combustion CC
	Vibrations affecting pre-combustion CC process; susceptible sensors and moving parts to damage
	Requirement for steady-state operation due to significant effect on energy conversion process
	Difficulty in adapting to fluctuating H_2_ demand during manoeuvres, necessitating additional measures like H_2_ buffers or dual-fuel engines

#### Research on onboard application of pre-combustion capture

So far, no literature sources have been found that deal with pre-incineration on seagoing ships. Only one example of pre-combustion capture was found, the HyMethShip concept [70]. This innovative concept integrates electromethanol energy storage, an on-board pre-combustion CC system and a dual-fuel combustion engine. The main objective of the concept is to create an almost closed loop for CO_2_ by integrating CC on-board. The captured CO_2_ is unloaded in the harbour and converted into electro-methanol, which is then used as fuel for the ship. An economic and life cycle analysis is also carried out in this context. This process is made possible by a pre-combustion process that converts electro-methanol into hydrogen and CO_2_. The assessment of this system extends from the wellhead to the ship's propeller, with a focus on ship operations in the North Sea until 2030 [70].

### Oxy-fuel combustion capture

#### Operating principle

**Fig. 7:**
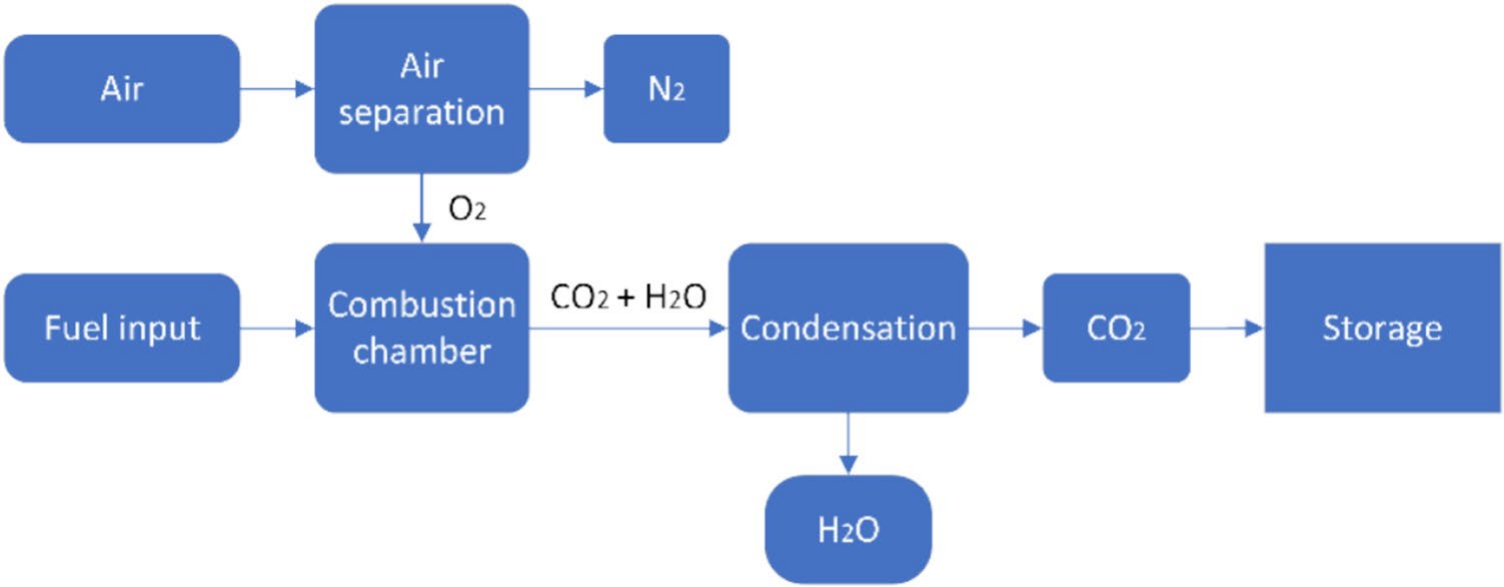
Schematic of oxy-fuel combustion capture

Oxyfuel combustion converts fossil fuels into CO_2_ and water vapour by burning them in the absence of atmospheric oxygen, assuming a fuel composition of CxHy without sulphur and nitrogen. Despite the simplicity of capturing CO_2_ and water vapour in theory with complete combustion, combustion is never 100% efficient in practise. In oxyfuel combustion, CO_2_ and water vapour are separated by condensation, as shown in Fig. 7. The success of this process depends on efficient separation of the water vapour, aiming for very low concentrations to enable effective CO_2_ compression and liquefaction. To achieve this, special drying systems are required due to the extremely low concentrations. Currently, oxyfuel combustion is used in the metallurgical industry and in coal-fired power plants [71]. Coal with the general formula CxHyNzSa may contain traces of other components such as ash, which, if not processed, will cause problems downstream and ultimately result in high maintenance costs. Despite its proven effectiveness in certain industries, interest and investment in oxyfuel combustion capture is limited in other sectors, as highlighted by Gür [72].

#### Advantages and disadvantages of oxy-fuel combustion capture for onboard application

The advantages and disadvantages pertinent to oxy-fuel combustion capture for onboard application are outlined in Table 3. This table serves as a reference point for the comparative study conducted in Sect. 7 of this study.

**Table 3:** Advantages and disadvantages of onboard oxy-fuel combustion capture

Advantages	Disadvantages
Easy capture of CO_2_ due to exhaust primarily containing CO_2_ and water vapour [73]	The Air Separation Unit (ASU) required for this process incurs high initial investment costs and demands substantial electricity [74]
Significant reduction in NOx emissions due to absence of nitrogen in combustion process [73]	Preventing air from entering combustion process and potential engine replacements are necessary but costly measures, hindering feasibility for retrofit applications [75]
Possibility of 0% NOx emissions with 100% pure oxygen	High risk of oxidising effect due to substantial amounts of highly concentrated oxygen, requiring protection of all metal surfaces against contact with O_2_ stream
No impact on ship's movement due to absence of free-moving liquids or solids	Limited fuel choice as all impurities are counterproductive to objectives and need to be addressed by aftertreatment
No need for heat for regeneration, potentially cost-effective in processes with limited waste heat [74]	Requirement for new engine materials to withstand high temperatures and difficulties in accommodating high-power demands of Air Separation Unit (ASU) in limited ship spaces [65]
	Generation of vibrations itself requiring a special engine for operation
	Safety concerns related to production and storage of oxygen add complexity to its marine application, necessitating a Hazard Identification (HAZID) and Hazard and Operability (HAZOP) study
	Technical defects in system may lead to loss of propulsion; inability to easily bypass compared to post-combustion capture

#### Research on onboard application of oxy-fuel combustion capture

There are currently no publications outlining concepts for the implementation of oxyfuel combustion on-board ships. However, a feasibility study conducted by Li et al. [76] examines the conversion of a conventional diesel-powered inland ship to an oxyfuel combustion system. While the study focuses on reducing oxygen consumption while maintaining the same energy output, it ignores crucial aspects such as space requirements, practicalities on-board and economic considerations. Interestingly, this concept opts to store the required oxygen in cylinders instead of producing it through an ASU [76]. Despite these challenges, oxyfuel combustion is promising if these hurdles can be overcome.

### Post-combustion capture

**Fig. 8:**
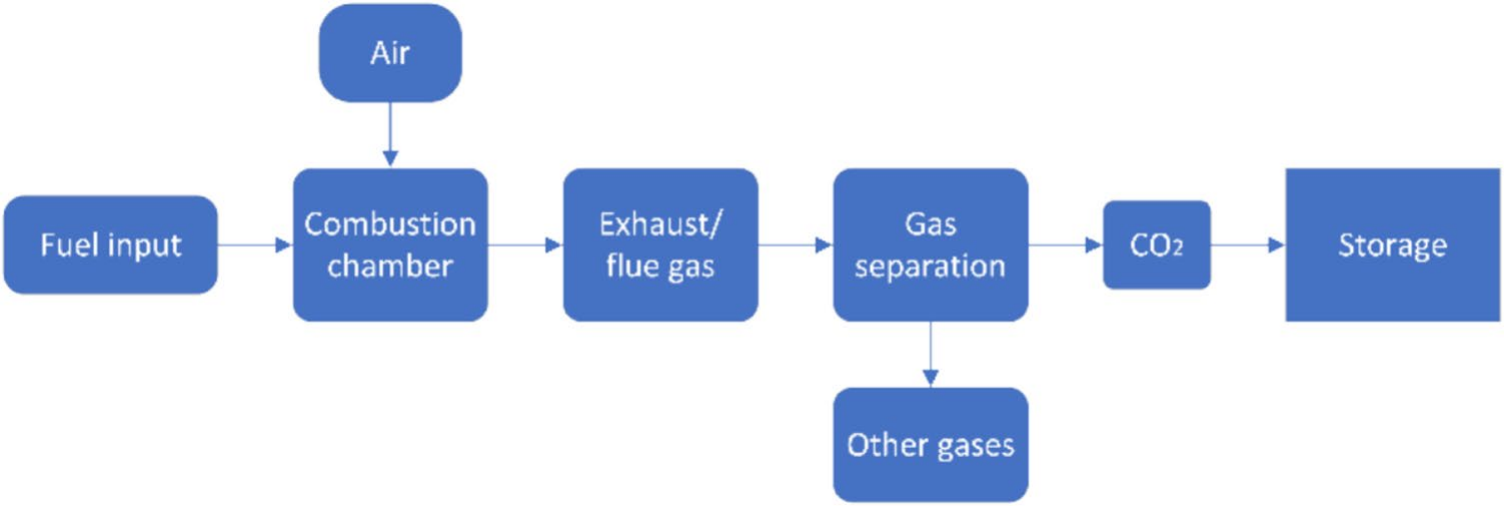
Schematic of post- combustion capture

Post-combustion CC involves the capture of CO_2_ from exhaust gases in flue gas environments after the combustion of carbonaceous fuels (as shown in Fig. 8). This method is often used in existing power plants [72]. In shipping, this technology can be used without the need to modify the engine or the entire system, only adjustments to the existing exhaust gas cleaning system are required. Since most marine engines have a turbocharger, the post-combustion capture system must comply with the engine manufacturer's specifications regarding minimum backpressure in order not to impair the performance of the turbocharger and the overall efficiency of the engine. Compared to pre-combustion capture and oxy-fuel combustion capture, post-combustion capture is easier to implement and requires relatively low fixed investment. It is also the most technically mature and practical method for the shipping industry. Post-combustion capture utilises various techniques, including chemical absorption, adsorption, membrane separation and cryogenic separation, which are explained in the following subsections.

#### Absorption by chemical solvents

Chemical absorption for CC represents a well-established and mature technology, particularly in the domain of post-combustion capture methods [77]. This approach involves utilising chemical solvents to capture CO_2_ emissions generated during industrial processes, making it a crucial component of GHG mitigation strategies. Significantly, large-scale applications such as coal-fired power plants have effectively implemented this technology for CO_2_ removal from their exhaust gases [78]. However, ongoing research efforts are dedicated to further improve the efficiency and effectiveness of this method [79].

##### Operating principle

Conventional absorption technology for CC consists of two main units: the absorber and the stripper. The absorber uses a lean absorption solvent to capture CO_2_ from the exhaust gas, while the stripper regenerates the solvent [77]. Effective mass transfer of CO_2_ between the gas and liquid phases is essential in both processes. The absorber, which often requires additional exhaust fans or blowers to overcome the increased pressure drop [77], is usually located in the flue gas stream and may include multiple columns of structured packing [75]. Conversely, the flue gases undergo pre-treatment to remove impurities before entering the absorber [77].

Impurities contained in the flue gas and the high exhaust gas heat, which can exceed temperatures of 300 °C and depends on the engine load and power, can lead to solvent degradation. Therefore, a pre-treatment system with a direct contact cooler is essential to cool the solvent down to room temperature [80]. During post-combustion CC, the lean absorbent flows in countercurrent with the exhaust gas and facilitates the removal of CO_2_ by absorption. The reversible chemical bonding between CO_2_ and the solvent enables efficient capture. The CO_2_-rich solvent is then channelled into the stripper unit, while the treated exhaust gas leaves the absorber with a reduced CO_2_ content.

**Fig. 9:**
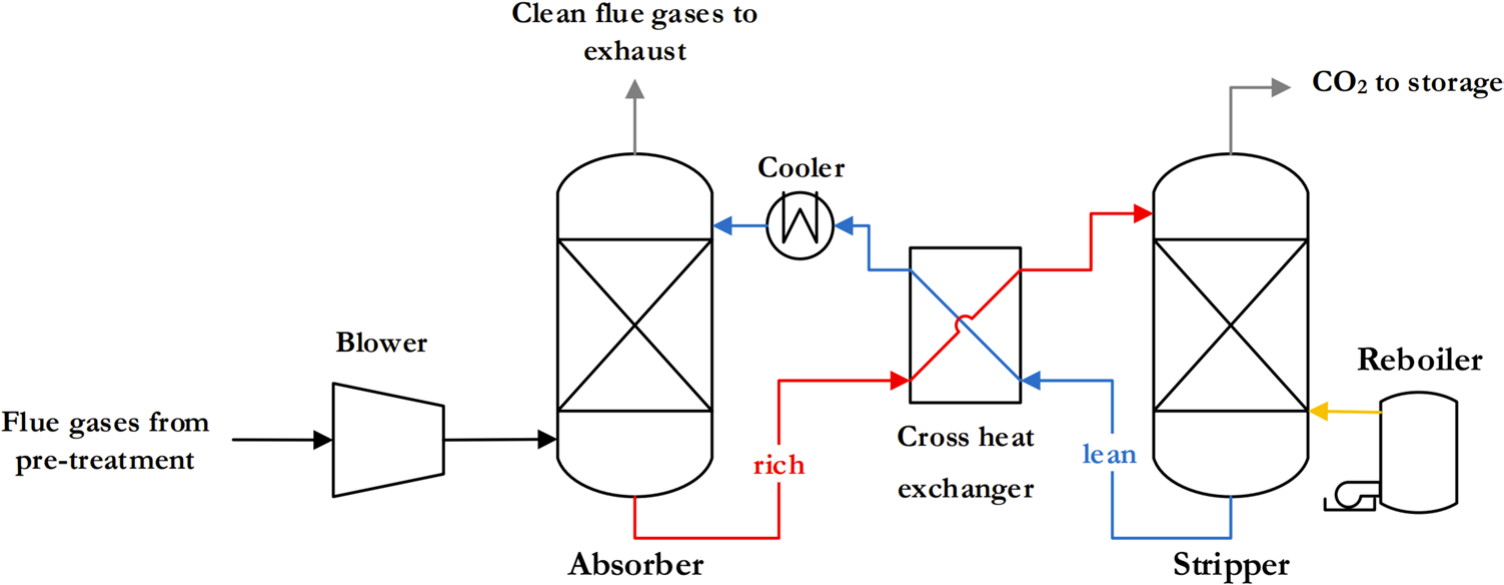
Schematic of chemical absorption process [73]

The absorber and stripper units are connected to each other, creating a cycle of lean and rich absorbent. A cross heat exchanger preheats the solvent before it enters the stripper to minimise heat loss. The stripper works in reverse to the absorber, releasing gaseous CO_2_ at the top and regenerating the solvent, which then returns to the absorber. The separated pure CO_2_ is pressurised and sent to special storage [77]. Figure 9 shows an illustrative diagram outlining the process flow to provide a better understanding of the technological arrangement.

##### Overview of different chemical solvents

In the field of post-combustion separation by chemical absorption, the use of a 30 wt% by weight solution of MEA (Monoethanolamine) is the best-known method. This particular method is often used in literature as a standard for comparing different CC technologies. The main reasons for the popularity of MEA are its high reactivity and cost-effectiveness in a wide range of flue gas conditions, as stated by Sreedhar et al. [77].

Apart from MEA, which is already commercially available, there has been intensive research into alternative chemical absorbents over the last ten years [81]. Many of these alternatives are based on amines, but ammonia-solvents, other aqueous liquids (water with solutes) and ionic liquids (liquids with salts) have also been explored. Each of these chemicals has its own advantages and disadvantages for certain applications and requires specific operating conditions, which can limit their applicability. In addition, researchers have explored the combination of different chemicals to enhance their benefits while minimising their drawbacks [77, 82]. Table 21 in the Appendix summarises some of these chemical solvents studied and provides an overview of their properties.

In this study, 26 reports/papers describing the use of CC on-board by chemical absorption were analysed. Among them, there is only one report on the CC-Ocean project [35], which describes the operation of a demo plant by the ship’s crew and assigns it a TRL 7 level for on-board operation. The other reports/papers listed are primarily simulation-based studies and case studies that could fall under TRL 2 and TRL 3. However, when looking at commercially available post-combustion CC plants, the most common and successful amine solvent for chemical absorption is 30 wt% MEA [77], which is categorised as successful at TRL 9. NH3 absorption technology is rated at TRL 6, based on successful pilot plant testing [83]. Concentrated piperazine (PZ) and its absorption capability reach TRL 6, with CO_2_ capture rates of 83.1–99.1% [84]. Ionic liquids for CC are at an early stage of research, with a TRL of 2–3 based on laboratory tests [85].

##### Advantages and disadvantages of chemical absorption for onboard application

Providing a comprehensive overview, Table 4 discusses the merits and demerits of onboard chemical absorption, serving as a basis for the comparative analysis in Sect. 7 of this study.

**Table 4:** Advantages and disadvantages of onboard chemical absorption

Advantages	Disadvantages
Remarkable adaptability for retrofitting into existing facilities without significant alterations to the power generation process [77]	Substantial energy demand required for solvent regeneration, posing a considerable challenge
Advanced maturity and widespread deployment across a wide range of flue gas applications [77]	Incurred energy penalties due to power needed for solvent pumps and exhaust stream blowers/fans, essential for overcoming pressure drop within the absorber [75]
Extensive real-world testing of components bolstering overall reliability	Toxicity and corrosiveness of certain solvents, along with their gradual degradation over time, posing operational risks
Continuous motion of absorber column may lead to relatively constant capture rate or slight increase, potentially enhancing overall absorption rate due to solvent redistribution [86]	Equipment demands considerable space and has substantial weight footprint, negatively impacting ship stability and limiting application in confined spaces [45] and [77]
Integration of SOx removal possible with ammonia-solvents (119)	Absorber diameters must be sized based on exhaust flow, which can affect the system’s reliability during ship manoeuvres
	Challenge in optimising CO2 absorption by solvent, requiring even distribution of exhaust gases across absorber column's diameter
	Impact on ship stability when tilting, causing gas flow to shift and necessitating additional gas distribution zones in absorber packing [87]
	Absorption processes using amine-solvents may work without pre-treatment but are impacted by degradation and emissions of hazardous by-products

##### Research on onboard application of CC by chemical absorption

CC by chemical absorption is proving to be the most advanced method for use on-board ships compared to other existing techniques. This is reflected not only in the abundance of research articles dealing with its application in the maritime sector, but also in various reports describing the processes and assessing the feasibility of CC by chemical absorption to reduce emissions from ships. This study identified 26 relevant articles and reports suggesting that CC by chemical absorption could help reduce carbon emissions in shipping. A summary of the key findings from these reports can be found in Table 22, where MEA, MDEA, DIPA and PZ stand for Mono-ethanolamine, N-Methyldiethanolamine, Diisopropanolamine and Piperazine, respectively.

In order to get the insights of the articles and reports mentioned in Table 22 (see Appendix), the major findings are grouped into the following categories:

*Effectiveness of different solvents*

- Various researchers have investigated different solvents for chemical absorption during carbon deposition. The solvents studied include MEA, PZ, MDEA, DIPA and ammonia.
- MEA is commonly studied and is consistently effective in various applications and ship types.
- Researchers such as Long et al. [88], Luo and Wang [44] and Ros et al. [86] found that MEA is a favoured solvent that achieves high CO2 removal efficiency.
- MEA is a commonly favoured option due to its effectiveness in scenarios ranging from diesel-powered ships to cargo and container ships.
- Ongoing efforts are aimed at finding optimal solutions tailored to specific operating contexts and ship types.

*Energy efficiency and operational considerations*

- Research into CC by chemical absorption in marine applications focuses not only on the environmental impact, but also on energy efficiency and operational aspects.
- Studies range from research into different solvents to innovative approaches such as the use of waste heat, the integration of additional gas turbines and the optimisation of the liquid-to-gas ratio.
- There are concerted efforts to improve the overall efficiency of CC processes on ships.
- Studies are looking at emissions reduction as well as capital and operating cost considerations and the integration of exhaust, heat and power systems.
- Notable achievements include a significant emissions reduction of 94% on a container ship by [89, 90] and a 14% reduction in the Energy Efficiency Design Index (EEDI) by Bayramoğlu [91] incorporating an Organic Rankine Cycle (ORC) system, demonstrating the tangible benefits of these efforts.

*Economic considerations and cost analysis*

- Economic assessments of on-board CC by chemical absorption in shipping emphasise the need for tailored strategies for different types of ships.
- The variability in cost-effectiveness is evident across the different methods.
- Key findings highlight the impact of capital expenditure and the importance of heat integration in reducing operating costs.
- Novel methods, such as the solidification of captured CO_2_ to realise a potential profit, and integrations such as cooling, heating and power systems, show economic benefits.
- Overall, the studies underline the potential for economic optimisation and efficiency improvements when implementing chemical absorption technologies on-board.

#### Adsorption by solid sorbents

During adsorption, atoms, molecules or ions from a gas or liquid attach themselves to the surface of an adsorbent and form an adsorbate film. Physisorption occurs due to van der Waals forces, while chemisorption occurs due to covalent bonds [75] and [92]. Chemisorption is slower as it requires electron transfer, making it less suitable for the uptake of large amounts of CO_2_, while physisorption offers a faster process and requires less energy to regenerate the sorbent [75] and [92]. In addition, physisorption is associated with a lower heat of adsorption, while chemisorption generally has a higher heat of adsorption [75] and [92].

##### Operating principle

**Fig. 10:**
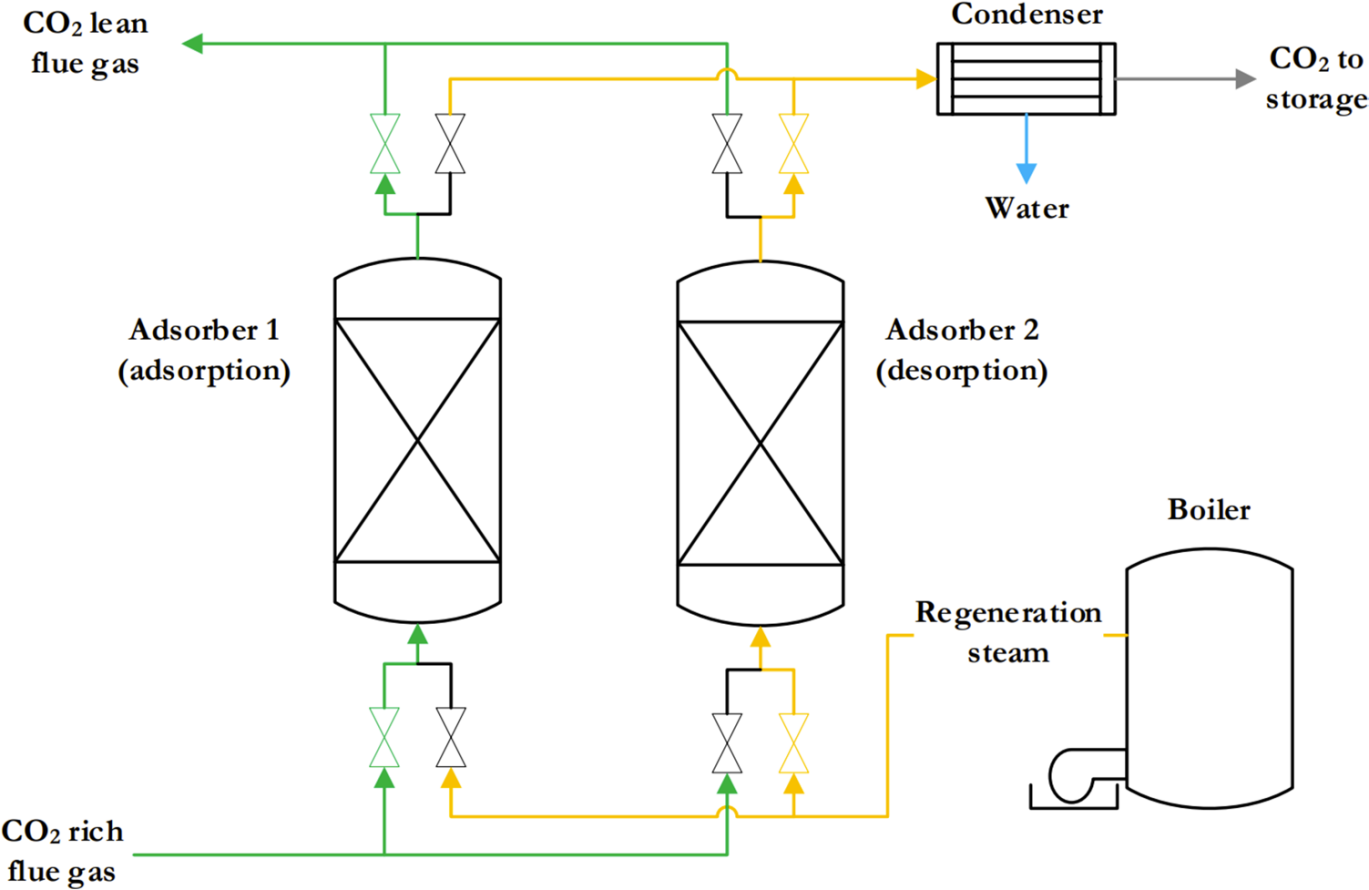
Schematic of a fixed bed adsorber using TSA [73]

The process of CO_2_ adsorption takes place in an adsorber system in which solid sorbents are arranged in columns. The effectiveness of this process, especially with dilute CO_2_ mixtures, is enhanced by physisorption, which involves the selective adsorption of CO_2_ molecules on the surface of the adsorbent [75]. Two main technologies for CC are fixed bed adsorbers and moving bed adsorbers. In fixed bed systems, the separation and desorption phases alternate cyclically within the same unit. Moving bed systems, on the other hand, feed the saturated adsorption material into a regeneration unit that provides a continuous off-gas stream without pressure drop issues. However, these systems face challenges in terms of wear and tear [75]. The adsorption system involves the use of auxiliary equipment such as blowers, fans and heat exchangers to facilitate the process. Different adsorption cycles, including pressure swing adsorption (PSA), vacuum swing adsorption (VSA) and temperature swing adsorption (TSA), can be used depending on factors such as gas volume and CO_2_ concentration. Each cycle comprises different phases, including adsorption, saturation, desorption and regeneration processes [75] and [92]. A simplified representation of a fixed bed adsorber with temperature swing adsorption (TSA) can be seen in Fig. 10, which provides a visual reference for the process [73].

In order to make CC by adsorption effective, certain important properties of the sorbent used are required. These properties include selectivity, capacity, ease of desorption, energy requirements, mechanical strength, chemical stability and cost [92] and [93]. Researchers are also investigating various adsorbents for CC. These include zeolites, metal–organic frameworks (MOF), porous silica, carbon-based materials (e.g. activated carbon) and solid amine-based materials [81].

##### Advantages and disadvantages of adsorption by solid sorbents for onboard application

In the context of onboard implementation, Table 5 sheds light on the advantages and disadvantages of adsorption by solid sorbents, laying the groundwork for the comparative study in Sect. 7 of this study.

**Table 5:** Advantages and disadvantages of onboard adsorption by solid sorbents

Advantages	Disadvantages
Physical adsorbents like zeolites and metal–organic frameworks (MOFs) exhibit high selectivity and capacity, making them effective for capturing CO_2_, especially at high pressures and low-temperatures [81]	Challenges in higher-temperature flue gases for physical adsorbents, leading to decreased capacity, especially at elevated temperatures [93]
Solid amine-based sorbents demonstrate a remarkable capability to capture high capacities of CO_2_ at low partial pressures, with superior CO_2_ selectivity when compared to physical adsorbents [81]	Susceptibility to oxidation and thermal degradation of amine adsorbents, limiting overall efficiency in CO_2_ capture
Versatility of adsorption technologies is underscored by their potential to capture CO_2_ directly from the air rather than from high-temperature flue gases [81]	Incompatibility of adsorbents with sulphur oxides (SOx) and nitrogen oxides (NOx) contaminants present in flue gas, potentially causing degradation [94]
	Efficiency of zeolites and MOFs is compromised by the presence of water vapor in flue gas because these adsorbents have a tendency to adsorb water before CO_2_ [95]
	Not suitable for flue gases of Internal Combustion Engines (ICEs) due to operational challenges and inefficiencies [81]
	Overall feasibility of CC by solid adsorption technology, when applied to flue gases from ICEs, currently deemed not feasible

##### Research on onboard application of CC by adsorption by solid sorbents

Only two articles were identified in this study, one of which focuses exclusively on the use of adsorption by physical solvents in marine applications. The second study focuses on road transport applications, but suggests that the technology could be extended to make it useful for capturing CO_2_ from marine exhaust. It is noteworthy that TSA is used in both articles.

Erto et al. [96] investigated the use of alumina-supported K_2_CO_3_ to capture CO_2_ from marine diesel engine exhaust. Their fixed bed adsorption process showed several advantages over solvent absorption, such as the use of non-hazardous materials, operational flexibility and the ability to capture CO_2_ at temperatures below 100 °C. Despite the need for a sulphur scrubber when using fuels with high sulphur content, the proposed method showed a CO_2_ reduction rate of 27.8 to 28.4% in a case study on a RoPax ferry. On the other hand, Sharma and Maréchal [97] proposed a concept for an energy self-sufficient CC and liquefaction system. Their technology is based on a TSA cycle using PPN-6-CH2-TETA as adsorption material and integrates a Rankine cycle, a heat pump and a CO_2_ compression and liquefaction unit. The system, originally designed for a lorry engine, achieved a capture rate of 90% in simulations. The researchers proposed to transfer the system to various combustion engines, including marine diesel engines.

The main information from both articles is summarised in Table 23 mentioned in Appendix.

#### Membrane technology

Research in the field of post-combustion of CC by membrane separation has experienced significant growth over the last two decades [98]. Membrane technologies, known for their ease of separation, are used in various industries, including the marine industry, where they are used in reverse osmosis plants for seawater desalination [99]. Various membrane configurations such as plate and frame membranes, spiral wound membranes and hollow fibre membranes are used, with two technologies standing out for CO_2_ removal: membrane gas separation (MGS) and membrane contactors (MC) [75] and [98].

##### Operating principle

MC technologies utilise microporous membranes to separate a CO_2_-rich gas stream from an amine-based liquid solvent and allow selective CO_2_ absorption as it permeates through the membrane. While the membrane facilitates this diffusion, the primary selectivity for CO_2_ removal is due to the properties of the solvent rather than the membrane itself [98]. The operating principle of MC technology is simple: flue gases enter on the gas side of the membrane, and a low CO_2_ stream exits the membrane through the gas outlet. On the sorbent side, the absorbing solvent, called permeate, circulates after CO_2_ absorption [100]. Like the absorption processes of chemical solvents, the liquid sorbent is regenerated. Hydrophobic membrane materials are used to prevent wetting and maintain mass transfer rates [98].

**Fig. 11:**
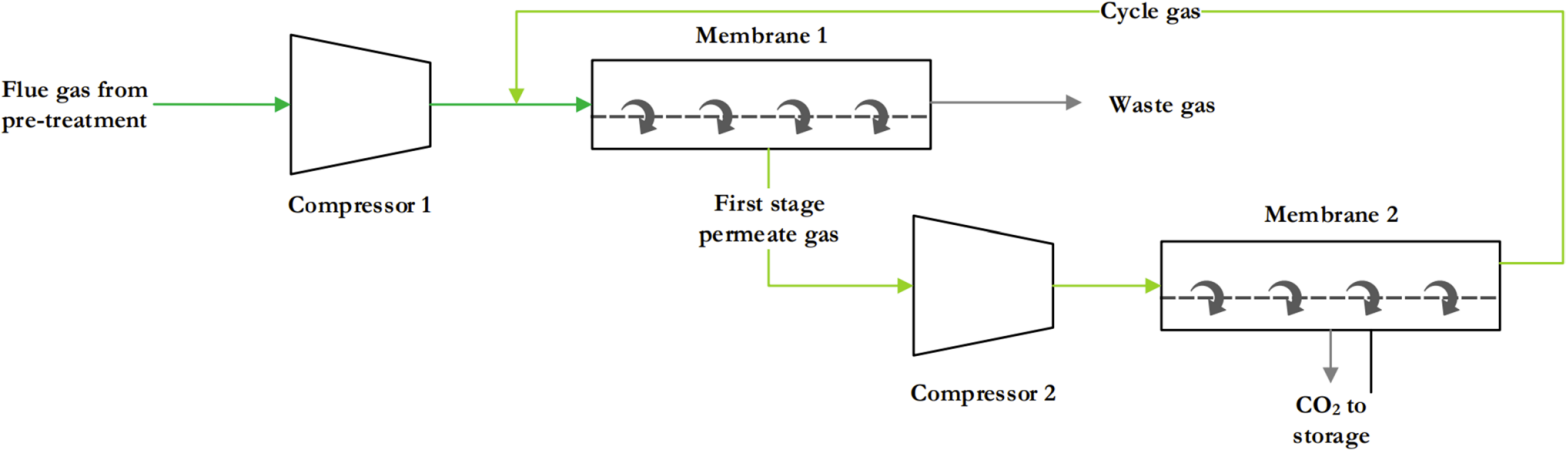
Schematic of a multi-stage membrane CO_2_ separation process [73]

The structure of MGS is similar to MC, but the permeate side is a gas phase. Compressors are used to increase the pressure of the feed gas before it enters the membrane unit, which increases the efficiency of CO_2_ removal while reducing energy requirements. Some systems use vacuum pumps on the permeate side of the membrane to achieve a similar increase in efficiency [101]. Recirculating the CO_2_-enriched permeate stream to a second or third membrane unit (as shown in Fig. 11) can increase capture rates, but also comes with additional costs, space requirements and energy consumption [98, 101]. In contrast to MC, MGS utilises denser, non-porous membranes, with CO_2_ selectivity depending solely on the membrane design, configuration and material [98]. Various mechanisms such as molecular sieving and solution diffusion facilitate CO_2_ capture in MGS [98].

##### Advantages and disadvantages of membrane technology for onboard application

Exploring the application of membrane technology onboard, Table 6 outlines both its benefits and drawbacks, crucial for the comparative assessment in Sect. 7 of this study.

**Table 6:** Advantages and disadvantages of onboard membrane technology

Advantages	Disadvantages
Polymer membranes widely used in CO_2_ capture from natural gases due to high selectivity, cost-effectiveness, and adaptability to various configurations [72]	Sensitivity to acidic gases and unsuitability for high-temperature exhaust conditions
Microporous inorganic membranes offer durability and corrosion resistance	Physical aging and plasticisation leading to reduction in membrane permeability and efficiency of separation process [75] and [98]
Highly compact design allowing for reduced size and flexible placement of the plant	Performance influenced by exhaust gas moisture
Reduced risk for ship personnel as the CC process can be bypassed in case of failure	Thorough pre-treatment of flue gas required to mitigate issues such as membrane fouling, degradation, and wetting phenomenon [75] and [98]
Presumed insignificant impact of ship movement on membrane separation arises from the absence of free-moving liquids or solids in the process	Higher operational costs for membrane gas separation systems due to energy-intensive nature of flue gas compression
Vibrations caused by ICE and weather conditions have less effect with systems having fewer moving parts like membrane technology	Weight added to overall system due to pre-treatment requirements
Opportunities for retrofitting into existing systems, showcasing potential in CC applications	Ongoing research needed to enhance material selectivity and permeability, focusing on surface engineering and incorporation of mixed materials [3, 4]

##### Research on onboard application of CC by membrane technology

The adoption of membrane technology for CC from flue gases, particularly in marine environments, is still in its nascent stages. There exist ongoing challenges that need to be addressed to enhance the durability of membranes. This is evident in the literature, as only one article was identified discussing the utilisation of membrane technology for CC in marine exhaust gases. The key findings from the reports/papers are summarized in Table 24 (see Appendix).

Oh et al. [102] considered a membrane-based CC and liquefaction system for LNG-fuelled ships to align with the IMO's 2050 GHG reduction targets. Compared to an amine-based system, the membrane approach shows competitive energy consumption (3.98 GJe/t_LCO2_ at 50 CO_2_/N_2_ selectivity). With improved selectivity (100 and 150), energy consumption decreases to 3.14 and 2.82 GJe/t_LCO2_, respectively. Moreover, the major equipment size decreases significantly when the permeance is 1000, 2000, and 3000 GPU (Gas Permeation Unit). On the other hand, Damartzis et al. [103] focussed on the post-combustion CO_2_ capture using modular membrane contactors, with solvent choice as a critical factor impacting efficiency and safety. A comprehensive review of solvents, considering key performance indicators (KPIs), revealed that, at that time, no solvent fully met all on-board operation objectives. While benchmark solvents like secondary amines showed compatibility and maturity, newer options like ionic liquids, though operationally superior, lacked maturity for on-board use. The study suggested accelerating research on advanced solvents for effective integration into maritime applications. Challenges in deriving a detailed quantitative ranking and the importance of studying solvent-membrane interactions were acknowledged, with a call for future research to develop indices correlating solvent properties with on-board operating characteristics for successful implementation.

#### Cryogenic CC

Cryogenic separation technology involves leveraging the phase changes of CO_2_ during extreme cooling in the flue gas stream produced by fuel combustion. Specifically, CO_2_ shifts from gas to solid directly, facilitating effective isolation of solid CO_2_ from the gas mixture. This process occurs at very low-temperatures and high pressures, capitalising on the discrepancies in gas boiling points. In the context of CO_2_ capture, the exhaust gas is typically chilled below the sublimation temperature of CO_2_ (− 100 to − 135 °C) while maintaining pressures of 10–20 MPa, enabling the separation of solid CO_2_ from other gases [72].

##### Operating principle

Cryogenic CC is a technology in which gaseous CO_2_ is converted into a solid state by cooling the flue gases at extremely low-temperatures. This process, known as desublimation, is crucial for the efficient capture of CO_2_ emissions. The system configurations for cryogenic CC may vary slightly depending on the approach chosen. In this study, two possible configurations for cryogenic CC are described: Compressed Flue Gas (CFG) and External Cooling Loop (ECL).

**Fig. 12:**
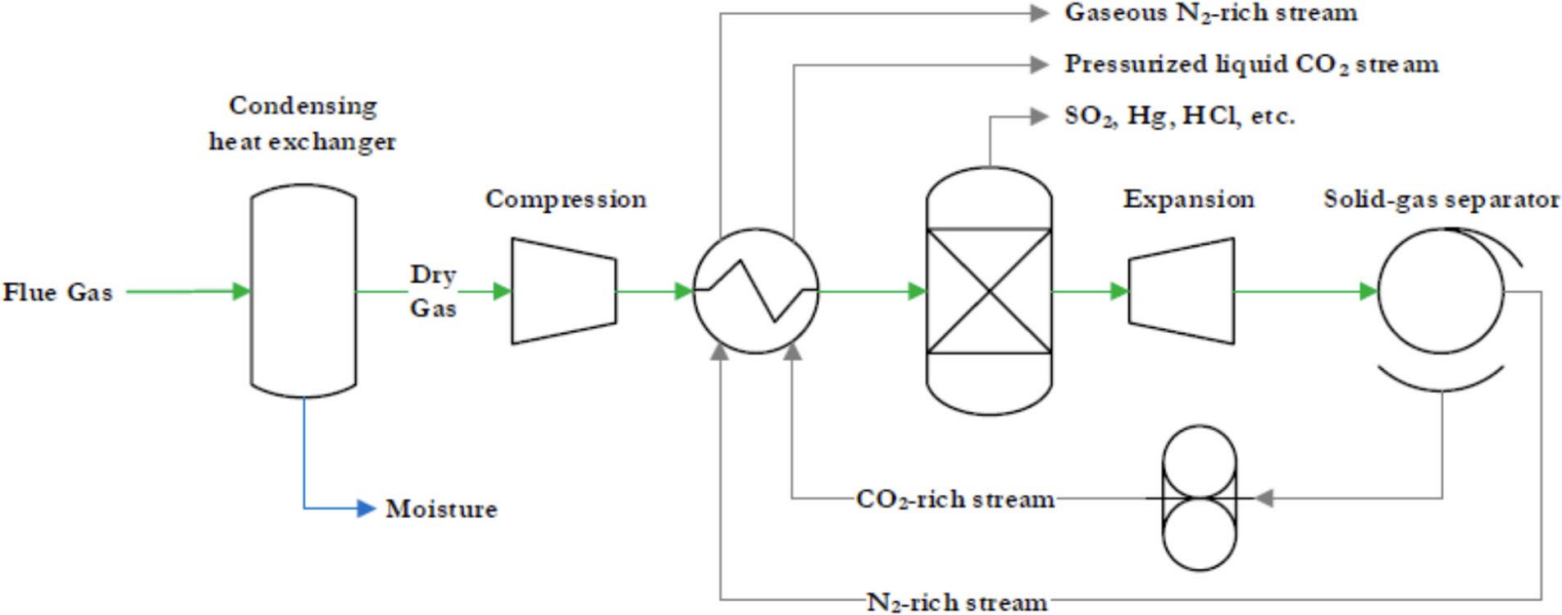
Schematic of a CFG system [104]

In the CFG system, the flue gases from the power plant pass through several stages: First, they pass through a dryer to remove water, then they are pressurised by a compressor and finally cooled in a heat exchanger, where the pressure is kept constant. Certain elements such as SO_2_, NO_2_, Hg, and HCl are effectively removed in condensed form by a highly efficient separator unit, as shown in Fig. 12.

After the separation process, the remaining flue gas consists mainly of N_2_ and CO_2_. This gas mixture is expanded via an expansion valve and cryogenically cooled, which solidifies the CO_2_. The solid CO_2_ and the remaining gaseous N_2_ are then separated in a solid–gas separator. The solid CO_2_ is pressurised and both streams are fed back into the heat exchanger to cool the incoming flue gases and simultaneously melt the solid CO_2_. At the exit of the system, the CO_2_ is in a pressurised liquid state suitable for storage or further use, while the remaining N_2_ gas stream can be released into the atmosphere at ambient pressure [104].

**Fig. 13:**
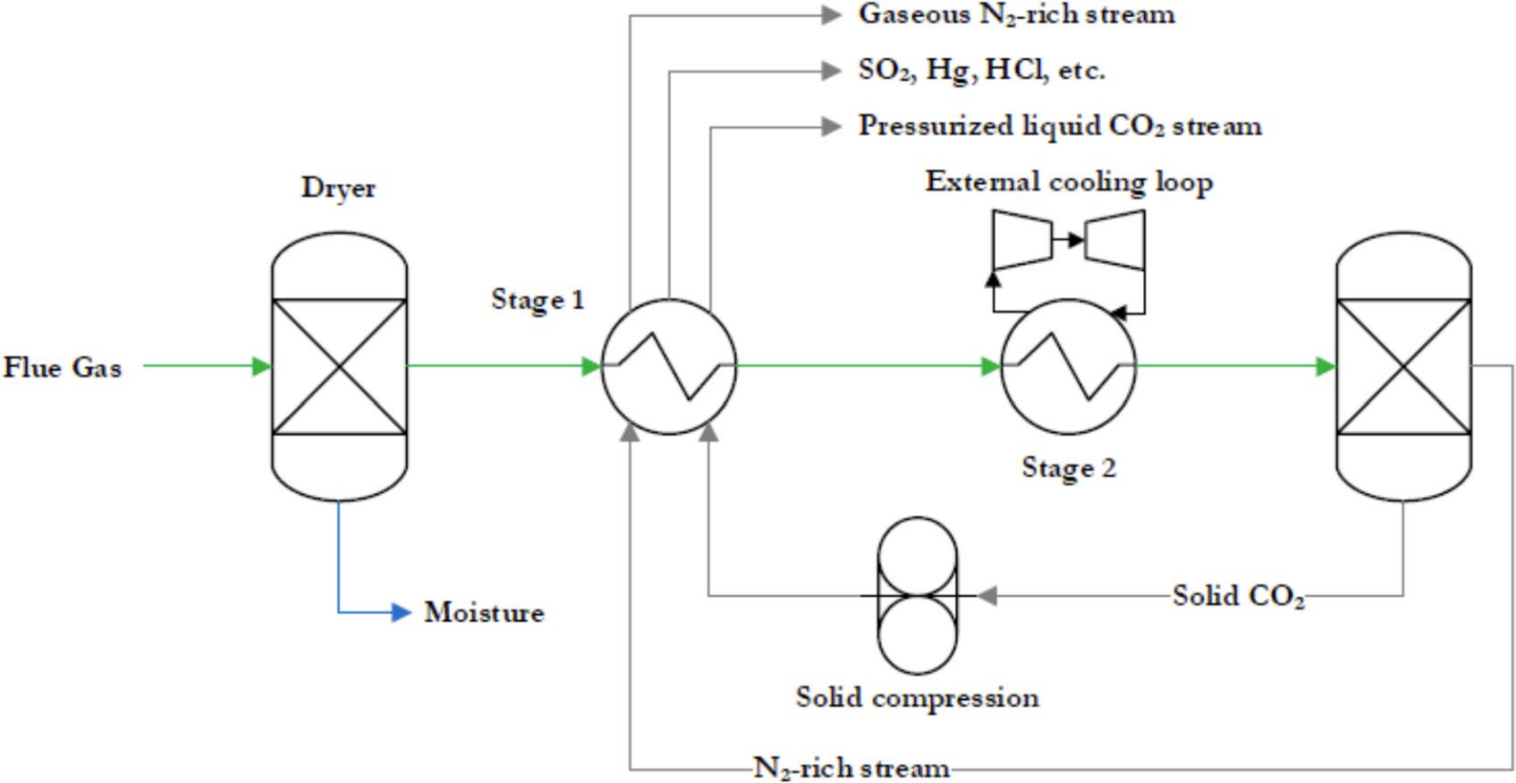
Schematic of a ECL system [105]

The ECL system is similar in design to the CFG system, but with one notable difference: it does not require compression of the flue gas. Instead, it usually includes a two-stage CO_2_ cooling process, a multi-flow heat exchanger and a desublimation heat exchanger. A simplified diagram of the ECL system is shown in Fig. 13.

At the beginning of the process, the flue gas stream is propelled by a blower and directed through a dryer unit to eliminate moisture. Subsequently, the dry flue gas proceeds to the multi-stream heat exchanger for precooling. The cooling required for this phase is obtained from an external cooling cycle, which operates through refrigerant compression and expansion. Moreover, extra cold energy is obtained by recirculating solid CO_2_ and liquid nitrogen from the outlet of the de-sublimating heat exchanger back into the multi-stream heat exchanger.

##### Advantages and disadvantages of cryogenic CC for onboard application

The advantages and drawbacks of employing cryogenic CC onboard are summarised in Table 7, offering a basis for comparison in Sect. 7 of this study.

**Table 7:** Advantages and disadvantages of onboard cryogenic CC

Advantages	Disadvantages
If the flue gas treatment facilities are already in place, this system can be retrofitted to any combustion process with minimal adjustments to the existing power plant	Inability to utilise waste heat from other processes, leading to additional energy requirements
Equipment takes up less space and has reduced impact on overall balance and stability of the ship	Production of waste heat, undesirable for onboard applications with excess waste heat
Less energy-consuming than chemical absorption technologies [104]	Formation of ice and accumulation of solid CO_2_ on heat exchanger surfaces may cause blockage, necessitating pre-removal of water vapor [106]
Pilot plant validation [107] has bolstered its reliability and contributed significantly to cost reduction efforts	Impact of ship’s movement on metal beads used for moving bed in A3C process [108]
Efficiently removes particles and pollutants from exhaust gases, including SOx, NOx, Hg, and HCl	Liquid form of separated CO_2_ saves energy for liquefaction and eco-friendly nature as it does not require chemical absorbers, making it suitable for marine and ecological environments
Achieves high purity and recovery rate of about 99.99% for captured CO_2_, surpassing other CC technologies that use harmful chemicals [109]	
Advanced cryogenic CC (A3C) process cools flue gas to 30 °C, removing pollutants and enabling the use of standard marine fuel oils [108]	
Potential bypass option available in case of system failure	

##### Research on onboard application of cryogenic CC

Cryogenic separation, which is often used for gas separation, is known for its high energy consumption. Research in the field of cryogenic CC aims to improve the energy efficiency of the corresponding processes. This study examines a report that assesses the feasibility and impact of introducing cryogenic separation in the shipping industry. It also discusses a project using cryogenic CC and the key findings are summarised in the Appendix, Table 25.

In 2020, Willson conducted an in-depth analysis of the application of the A3C process for ships, focussing on its technical and economic aspects. The A3C technology developed by PMW Technology, which is currently at TRL 3 to 4, has shown promising cost reductions in land-based applications, which makes it interesting for use at sea. The core of the A3C process consists of two stages with a unique moving bed of metal beads that enables efficient CO_2_ capture in a compact design. Importantly, the A3C process optimises energy efficiency using waste heat to vaporise solid CO_2_ on the metal spheres, significantly reducing the overall energy requirements of the cryogenic system. In case studies conducted on a car carrier and a RoPax ferry, CO_2_ reduction, ship stability and economic factors were evaluated. The results showed competitive performance, especially when A3C was used for all LNG-fuelled engines, indicating its potential as a cost-effective alternative for CC in the maritime industry.

In addition, the decarbonICE project [37] actively explored conceptual designs for onboard CC systems. This initiative aimed to store captured CO_2_ in the form of dry ice, which is moulded into Carbon Descent Vehicles and released into the sea for safe storage in seabed sediments. The project addressed various aspects including environmental concerns, technical feasibility, cost analysis, safety and risk assessment. If ships equipped with the decarbonICE technology use carbon–neutral biofuels, the system has the potential to achieve carbon–neutral shipping, which is a significant step towards sustainable maritime operations.

## Onboard storage of CO_2_

The storage of CO_2_ can be categorised into long-term and temporary storage. Long-term storage is focused on reducing atmospheric CO_2_ levels and includes geological storage, ocean storage, mineral carbonation, and industrial uses. On the other hand, temporary storage involves transporting CO_2_ from capture to the final storage point. During onboard CC, the captured CO_2_ needs temporary storage. This study examines the possibilities of temporary onboard CO_2_ storage. In this regard, various approaches can be considered depending on the pressure and temperature of CO_2_. The viable options for storing CO_2_ onboard include a supercritical state, gaseous state, solid state, or liquid state.

### Gaseous storage

The paper [110] suggests that storing CO_2_ in gaseous form is impractical due to the considerable volume it would occupy, although it requires less pressurisation and cooling compared to other phases. In addition, the gaseous form is the state with the lowest density of CO_2_. It is 172 kg/m^3^ at 30 °C and 60 bar, while the density of supercritical CO_2_ at 35 °C and 125 bar is 757 kg/m^3^, the density of liquid CO_2_ at − 15 °C and 30 bar is 1011 kg/m^3^ and the density of solid CO_2_ at − 80 °C and 1 bar is 1562 kg/m^3^ [111]. It is therefore not used for the transport of large quantities of CO_2_. However, the feasibility of storing CO_2_ in a gaseous state depends on the operational profile, the general layout of the ship as well as the cost and energy requirements.

### Storage at supercritical phase

**Fig. 14:**
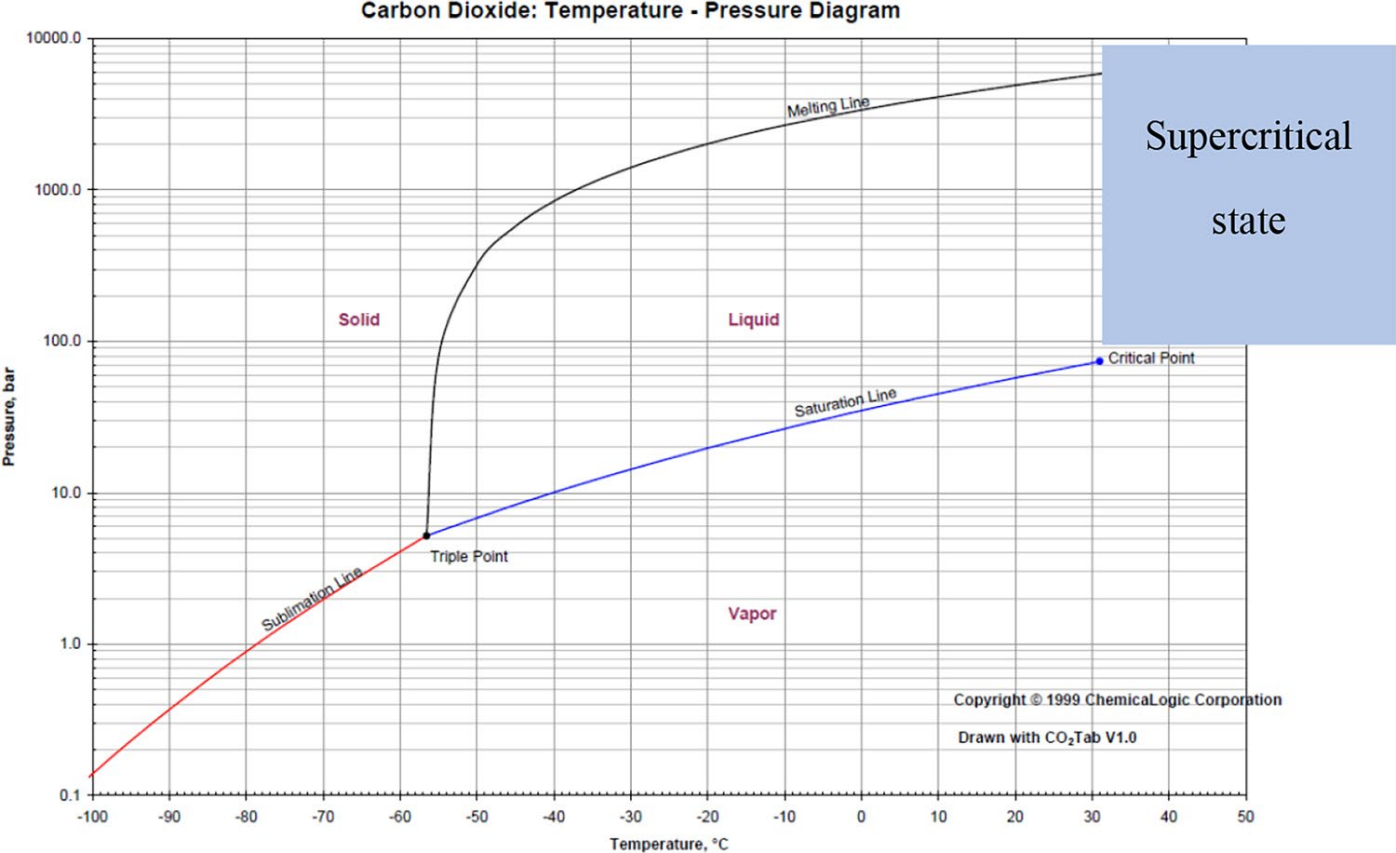
Phase diagram of CO_2_ [112]

The supercritical phase is attained by compressing CO_2_ above 73 bar (critical pressure) and beyond 31.1 °C (critical temperature), as illustrated in Fig. 14. This phase, known as the supercritical fluid phase, is the favoured state for pipeline transportation due to its higher density compared to compressed gas. For pipeline operations, the typical operating pressure is above 96 bars, chosen for its cost-effectiveness. Pressures lower than 96 bars may result in two-phase flows, which are preferably avoided (refer to Fig. 14).

### Solid storage

To facilitate the storage of CO_2_ in a solid state, two primary approaches can be considered. In the first method, the temperature of the CO_2_ is lowered to − 78 °C, causing it to solidify under atmospheric pressure conditions (see Fig. 14). At this specific temperature and pressure, the enthalpy of sublimation of the gas is given as 573 kJ/kg. This means that in addition to cooling the gas to − 78 °C, an additional 573 kJ of energy per kilogramme of CO_2_ must be extracted to initiate solidification—a process that requires a considerable amount of energy [112].

Another possibility for solid CO_2_ storage is the chemical binding of CO_2_ to another substance. Although some research suggests that this method is suitable for CO_2_ storage on-board ships [65] and Zhou & Wang 2014, the technology is still at the development stage and is mainly limited to laboratory experiments. It is not yet mature enough for widespread commercial application. In addition, the implementation of this chemical binding approach would require the presence of the intended substance on-board, which would lead to a significant increase in the overall weight of the ship.

A particular challenge associated with the storage of CO_2_ in a solid state—whether by refrigeration or chemical sequestration—is the need for a robust system capable of handling solid CO_2_ effectively on ships. In the case of refrigerated CO_2_, the implementation of a closed system is crucial to prevent the sublimation of CO_2_. Such sublimation could cause air to escape from the engine room, posing a serious risk of crew asphyxiation. The development and implementation of such a system represents a remarkable and complex challenge in the context of maritime operations.

### Liquid storage

Storing CO_2_ in liquid form is advantageous because it is easy to handle with pumps. In addition, the volume required to store CO_2_ is significantly lower due to the density of the liquid form. There are several strategies for this, each differing in the temperature and pressure at which storage takes place. The triple point of CO_2_, which is 5.18 bar and − 56.6 °C, means that CO_2_ only exists as a gas or solid at atmospheric pressure. To keep it in liquid form, a pressure of at least 5.18 bar is required. However, storing CO_2_ near its triple point carries the risk of solid CO_2_ formation, which could clog pipelines and be difficult to remove from storage tanks. It is therefore recommended to store CO_2_ well above its triple point.

This study identifies two relevant articles that describe the ideal temperature and pressure conditions for liquid storage and transport of CO_2_ on-board under specific conditions. A summarised overview of the results can be found in the Appendix, Table 26.

Seo et al. [112] proposed ship-based CCS chains with different CO_2_ liquefaction pressures and evaluated the life cycle cost (LCC) to determine the optimal pressure. Seven pressures were considered in this study, ranging from 5.18 to 73.8 bar. The chain consisted of five modules: liquefaction system, storage tanks, CO_2_ carrier, intermediate storage tanks and pumping system. In terms of LCC, which includes both CAPEX and OPEX, the results showed that 15 bar was the optimum pressure. As the pressure increased, the liquefaction and pumping costs decreased, while the storage and CO_2_ carrier costs increased. In particular, the liquefaction system dominated the LCC, while the pumping system contributed the least. The sensitivity analysis confirmed that 15 bar is optimal, regardless of disposal volume, distance, methodological uncertainty, and unit electricity costs.

On the other hand, Bjerketvedt et al. [113] analysed the historical use of ship infrastructure for CC and storage in Norwegian industry. Using a mixed-integer multi-period model, they optimised the transport investments, routing and transport portfolio to connect nine facilities to the Northern Light Initiative. The optimised portfolio resulted in a cost reduction of 12 compared to independent transport chains. While the 7-bar transport was cheaper than the 15-bar transport, the technological maturity of the 7-bar transport was a disadvantage. The model identified two cost-optimal chains: the 15-bar chain with an average cost of €32.4/tonne and the 7-bar chain with an average cost of €25.4/tonne. The net present value (NPV) analysis determined the conditions for an economic retrofit of the 15-bar chain, with the break-even point at a resale price of the ship of 60% and the retrofit costs at 40% of the investment.

Regarding storage pressure, while low-pressure systems, such as the 7-bar transport, are generally easier to manage and less energy-intensive, they face limitations in terms of the volume of CO_2_ they can store, requiring larger space on ships. High-pressure systems, though more energy-intensive, are better suited for compact storage. Each system has its own advantages and disadvantages, and the choice between low and high pressure depends on factors such as space availability, operational costs, and technical maturity of the systems.

In summary, the studies showed that 15 bar [112] is the optimum liquefaction pressure, which creates a balance between reduced liquefaction and pumping costs. Shipping at 7 bar was cheaper [113], but concerns were raised about technological maturity. The NPV analysis provided the conditions for an economic retrofit of the 15 bar [113] chain and emphasised the trade-offs between cost efficiency and technological considerations when implementing CC and storage.

### Containment systems for CO_2_ shipping

As far as the phases of CO_2_ are concerned, the gaseous and supercritical phases are not normally used for storage on-board ships. Instead, CO_2_ is usually transported in a liquefied state, which requires special containment systems designed to keep the gas at specific temperature and pressure conditions to prevent vaporisation. Supercritical CO_2_ tends to be transported in pipelines due to its specific temperature and pressure conditions, which are generally not suitable for storage on-board ships.

In CO_2_ shipping, special containment systems are crucial for the safe and efficient transport of liquefied carbon dioxide. These systems are designed to keep CO_2_ in its liquid state at low-temperature and high pressure while complying with regulatory requirements and minimising the potential risks associated with handling liquefied gases. Several established containment systems are currently used in the industry for CO_2_ shipping, including:

#### Type C tank systems

The International Code for the Construction and Equipment of Ships Carrying Liquefied Gases in Bulk [114] prescribes Type C tank systems, which are commonly used in CO_2_ carriers. However, the adaptation of these tank systems to the specific characteristics of liquefied CO_2_, such as its higher specific gravity compared to gases such as LPG, must be carefully considered.

#### Membrane tank systems

Another popular containment system is the membrane tank, typically used for the transport of liquefied natural gas (LNG) and also applicable for CO_2_. This system is characterised by its thin, flexible membrane that forms a lightweight and cost-effective solution. However, these tanks require specific structural designs and insulation systems to maintain the required low-temperatures.

#### Spherical tanks

Spherical tanks, also known as pressure vessels, are sometimes used for the storage and transport of CO_2_. These systems offer high strength and are suitable for storing CO_2_ under high pressures. Their spherical shape allows for better distribution of stress and makes them a reliable choice for safely storing liquefied CO_2_.

According to Tanaka et al. [115], the following aspects must be considered in containment systems for CO_2_ transport.

#### Selection of materials

The selection of materials for the containment system is of crucial importance for its effectiveness and safety. Materials with high strength and suitable low-temperature properties are preferred, such as heat-treated steel, low-temperature steel and Ni steel. The choice of material depends on various factors, including the specific properties of the CO_2_ being transported and the structural requirements of the ship.

#### Structural analysis

A detailed numerical analysis is required to assess the structural integrity of the containment system, including the tank support structures. This analysis ensures that the containment system can withstand the pressure and stresses during transport and handling.

#### Reduction of sloshing

Sloshing, the oscillation of the liquid in the tank, poses a significant risk to the integrity of the containment system. CFD (Computational Fluid Dynamics) calculations are good enough to predict the sloshing behaviour under different sea conditions. Measures are then taken to minimise the potential damage from sloshing, e.g. reinforcing the tank structures and securing the internal fittings.

#### Dynamic process simulations

Dynamic process simulations are required to anticipate changes in CO_2_ conditions during transport and loading and unloading. By simulating different scenarios, potential risks, such as reaching the triple point in pressure or temperature, can be recognised and mitigated by taking appropriate measures.

Overall, containment systems for CO_2_ transport must strike a balance between safety, efficiency and reliability. Material selection, structural analysis, sloshing mitigation and dynamic simulations are integral components to ensure the successful transport of liquefied CO_2_.

### CO_2_ storage in LNG fuel tank

To reduce the costs associated with onboard CC, it is worth considering the possibility of storing the captured CO_2_ in the ship's existing LNG tanks when it is not needed. In this context, the term ship refers to an LNG carrier or any other ship that uses LNG as fuel. This strategy could lead to cost savings on the initial investment while increasing the available cargo space on the ship [116].

However, several technical considerations arise when examining the potential for CO_2_ storage in the ship's LNG tanks, as highlighted by Van Den Akker [116], 5which are mentioned below:

#### Pressure and temperature compatibility

LNG is usually stored at a pressure of up to 10 bar, while CO_2_ requires a minimum pressure of around 7 bar to prevent solidification. For optimum liquefaction energy, a higher pressure of 16–18 bar is required, which necessitates modifications to the tanks to cope with these pressures.

#### Temperature challenges

LNG is stored at around − 160 °C, while liquefied CO_2_ maintains a temperature of around − 27 °C. This temperature difference can cause fatigue. This temperature difference can lead to fatigue problems. A possible solution is to store LNG at a higher pressure and temperature, e.g. 15 bar and − 120 °C, to minimise temperature fluctuations.

#### Contamination and flushing

To avoid contamination and possible clogging of the fuel lines, a thorough cleaning of the tanks from CO_2_ before refuelling with LNG is essential.

#### Fuel residues and solidification

Tanks should be emptied as much as possible before filling with CO_2_, as any remaining LNG will become unusable for propulsion once CO_2_ enters. In addition, a significant amount of LNG in the tank could cause the CO_2_ to solidify during filling, vaporising the LNG and creating high tank pressure.

#### Density considerations

Liquid CO_2_ has a density of more than 1 tonne/m^3^ at 18 bar and − 24 °C, which is more than twice the density of LNG (about 0.45 tonnes/m^3^). Consequently, the tank and its supporting structure must be sufficiently robust to cope with the increased loads associated with this higher density.

The authors have limited information on the use of tanks for both LNG and CO_2_. While some attention has been paid to this topic in the context of CO_2_ transport, there is a lack of actual research on the subject. As a result, research into this area could prove beneficial for LNG carriers looking to integrate CCS. Areas that need to be researched include the process of emptying CO_2_ from the LNG tank, the possible replacement of the LNG tank and the methods of monitoring the LNG level to ensure that it is low enough for the tank to serve as a temporary CO_2_ storage facility.

### Storing captured CO_2_ for CO_2_ carrier

If the ship's cargo is CO_2,_ mixing the captured CO_2_ with the cargo would depend on the specific storage and handling requirements of both the CO_2_ and the cargo. In general, mixing captured CO_2_ with the cargo might not be ideal unless both can be stored and transported under similar conditions. Here are some key factors to consider:

#### Storage conditions

CO_2_ for transport typically needs to be stored under specific conditions, such as being liquefied or kept at high pressure, to ensure it remains stable during transit. If the cargo is different from CO_2_ (e.g. chemicals or other goods), its storage conditions may not be compatible with those needed for CO₂, leading to potential safety or operational issues.

#### Purity and quality control

Captured CO_2_ from a ship’s engine exhaust may not be of the same purity as the CO_2_ cargo (which is usually captured and processed for sale or use in other industries). Mixing impure CO_2_ with commercial CO_2_ could degrade its quality, which could have implications for its future use or sale.

#### Logistics and separation

From a logistical perspective, mixing CO_2_ with the cargo could complicate handling, as it would require systems to separate the CO_2_ from other materials if necessary. Captured CO_2_ would typically need to be isolated for storage or eventual disposal, which could require dedicated tanks or containment systems on the ship.

#### Safety and regulation

Regulatory and safety considerations would likely prevent the direct mixing of CO_2_ with other cargos unless it is specifically designed and approved for that purpose. For example, the IMO and other regulatory bodies have stringent rules for the transport of gases and hazardous materials, which might limit the mixing of CO_2_ with certain types of cargo.

In short, while it may be technically feasible to store CO_2_ in the same vessel as the cargo, it is unlikely to be mixed unless both the cargo and the CO_2_ can be stored under compatible conditions. For most CCS systems, CO_2_ would likely be stored separately from the cargo to ensure safety, quality control, and regulatory compliance.

## Implementation challenges for onboard CC

This section describes the challenges associated with the integration of CC technologies on land on-board. Successfully adapting these technologies for shipping requires overcoming unique hurdles, including spatial constraints, weight limitations and the need for increased system robustness to withstand maritime conditions. In addition, seamless integration into existing ship designs, compliance with safety standards and managing potential operational disruptions present additional complexities in the maritime environment. Below you will find a list of the relevant challenges for the consideration of CC on-board, which have been identified through a literature review.

### Compatibility with marine ICEs

CC technology must be compatible with marine combustion systems and capable of withstanding the high temperatures found in the exhaust gases of combustion engines. Since most marine engines are turbocharged, the technology must also meet the minimum backpressure levels set by the engine manufacturer to avoid negative effects on turbocharger performance and overall engine efficiency. This challenge applies to all CC technologies, including oxy-fuel combustion, post-combustion methods (such as chemical absorption or cryogenic separation), and membrane separation. Regardless of the specific technology, the primary concern is whether it allows the engine to operate within the required backpressure range without compromising engine performance.

### Ship safety and stability

The installation of CC technologies on the ship may have an impact on safety and stability and raise concerns about potential hazards or increased risks to the crew. In addition, the impact of a CC system failure on the ship must be considered. The installation of these systems also affects the metacentric height (GM) and therefore has an impact on overall stability.

### Ship movements and vibrations

The movement of the ship at sea can have a negative impact on CC technology. Vibrations on-board have the potential to reduce the efficiency of the CC system.

### Engine load variation

A ship's energy requirements change during different operating modes, leading to fluctuations in engine load and consequently fluctuations in fuel consumption and CO_2_ mass flow generation. During manoeuvres, the energy demand can change rapidly. The OCCS system must adapt to these fluctuations by either providing sufficient fuel for the required energy during pre-combustion or effectively capturing the CO_2_ from a variable exhaust gas flow and variable exhaust gas temperatures during post-combustion.

### Impurity tolerance

The technology can be affected by impurities in the fuel/exhaust gas stream. Certain solvents can be susceptible to impurities such as sulphur, particulate matter or traces of methane. The presence of these compounds in the exhaust gas could affect capture efficiency. Furthermore, in the compression and liquefaction of CO_2_, the permissible water vapour content is limited to less than 50 ppmv [117].

### Maturity level

While CC technology is well-established on land, there are few demonstration cases for CC technology at sea, especially at low capture rates. The development of efficient and compact CC technologies on-board ships that can be easily integrated into the current ship design while ensuring safety and reliability is a major challenge.

### Space constraints

The main function of a ship is to transport goods, and the value of a ship is closely linked to the space available for cargo. Maximising cargo space is crucial for higher revenues. The less space taken up by the installation of the CC unit and associated tanks for intermediate CO_2_ storage, the more cargo the ship can carry. Retrofitting or installing CC systems on-board is a challenge due to limited space and risks compromising cargo capacity or crew accommodation.

### Onboard energy utilisation

The energy required to operate a system on a ship must be generated onboard, i.e. the electricity required for the CC technologies must come from onboard generators. This is at the expense of fuel. A higher energy requirement leads to higher operating costs, including fuel and maintenance.

### Capture rate

It is important to assess the achievable CO_2_ capture rate, considering the additional energy consumption.

### Additional weight

Ships are designed to carry a certain weight of cargo, known as deadweight tonnage (DWT). The installation of a CC system, the intermediate storage of the captured CO_2_ and the required chemicals increase the weight and thus reduce the DWT.

### Cost implications

Ship owners attach great importance to the initial investment costs. Increased investment costs could make the introduction of such technology economically unfeasible and extend the amortisation period. The operating costs result primarily from energy consumption and consumables. The lower the operating costs, the more cost-effective it is to operate a CC system.

## Comparative study amongst different CC technologies to address the challenges

This section attempts to identify the most promising of the technologies discussed in the previous sections for shipboard applications, specifically addressing the challenges described in Sect. 6. While different articles focus on different CC technologies, a comparative evaluation of these technologies to assess their relative effectiveness is lacking. Furthermore, the results are predominantly drawn from different case studies, which further complicates the determination of the most appropriate CC technology on-board. In this context, the authors endeavour to compare the CC technologies discussed in Sect. 4, assess their potential to address the challenges described and rank them accordingly. The CC technologies are first assessed against the following five challenges that justify their feasibility for installation on-board ships.

- Compatibility with Internal Combustion Engines (ICEs).
- Impact of CC technology on the ship's safety and stability.
- Influence of the ship's motion and vibration on CC technology performance.
- Ability to handle engine load variation.
- Tolerance of impurities in the fuel/exhaust.

The three best CC technologies that fulfil all five criteria are then evaluated at the next level based on the other challenges mentioned in Sect. 6. In this study, a qualitative ranking scale is used to compare the technologies. This approach involves subjective judgements, assessment of "soft" or non-quantifiable data and engagement with intangible information, which presents a challenge in measurement. As part of the research, reports/papers are comprehensively reviewed to describe the strengths and weaknesses of each CC technology and place them in the context of specific challenges to determine their comparative ranking. Given the subjectivity of the ranking process, careful justifications are provided prior to ranking the CC technologies, especially when it comes to each individual challenge. The same type of ranking methodology is also used to create risk matrices for newly introduced technologies on-board ships [118]. The following ranks are used in this study:

*Highest*: Outstanding potential in addressing the challenge.

*High*: Strong potential in addressing the challenge.

*Moderate*: Reasonable potential with room for improvement in addressing the challenge.

*Low*: Limited potential with significant constraints.

*Lowest*: Minimal potential, severe limitations, consider alternatives.

It is important to underscore that the rankings assigned in this study to each CC technology are based on their potential to address specific challenges, evaluated through an extensive literature review. It is crucial to note that these rankings are not definitive and are subjective. Nonetheless, significant disparities in the assigned ranks are not expected.

### Initial stage assessment

#### Compatible to ICEs

Given the likelihood that newbuild ships will primarily incorporate ICEs for energy conversion in the foreseeable future, it becomes crucial for CC technologies to be compatible with these marine ICEs as well as need to adhere to the minimum backpressure specifications set by the engine manufacturer. A comprehensive literature review has informed the development of Table 8, which identifies CC technologies compatible with ICEs. These technologies are selected for further investigation, aligning with the overarching goal of satisfying additional criteria and ensuring seamless integration with modern marine energy conversion systems.

**Table 8:** Compatible to ICEs

CC method	Compatibility to ICE	Supported reports/papers	Findings	No of supported reports/papers
Pre-combustion	Yes	[70]	The pre-combustion system in the HyMethShip concept is compatible with ICE	#1
Oxyfuel combustion	Yes	[75, 76]	Explores the adaptation of a conventional diesel engine to oxy-fuel combustion, however, when oxy-fuel combustion is first implemented, the brake power of the engine initially decreases compared to conventional air combustion	#2
Chemical absorption by NH_3_/MEA/PZ/Blend	Yes	[26, 44–46, 61, 86, 88–91, 94, 116, 119–125, 137]	Chemical absorption technologies, particularly those utilising solvents like NH_3_, MEA, PZ, or blends, are compatible with ICE	#20
Chemical absorption by NaOH and CaO	Yes	[136]	Chemical absorption using NaOH and CaO for CO_2_ capture is compatible with ICE but need to work on how to optimise regeneration efficiency and material stability	#1
Membrane separation	Yes	[102, 103]	Membrane separation is compatible with ICE, offering a promising method for selective gas separation with potential for integration in carbon capture systems	#2
Cryogenic separation	Yes	[37, 108]	Cryogenic separation is compatible with ICE, offering a feasible method to separate CO_2_ from other gases based on their different boiling points	#2
Adsorption by solid sorbents (in general), except alumina-supported K_2_CO_3_	No	[81, 92]	Adsorption by solid sorbents, except for alumina-supported K_2_CO_3_, is not considered compatible with ICE, which highlight limitations in integrating solid sorbent-based CO_2_ capture technologies into ICE systems	#2

In accordance with Table 8, five CC technologies—Pre-combustion, Oxyfuel combustion, Post-combustion Chemical absorption, Membrane technology, and Cryogenic separation—have been selected for in-depth assessments. Notably, adsorption by solid sorbent is excluded from consideration, as the literature review has determined its infeasibility for use in higher-temperature flue gases of ICEs in the context of marine applications.

#### Ship safety and stability

Assessing the impact of CC technologies on the safety and stability of ships requires a thorough consideration of various factors. These considerations can be broadly categorised into safety considerations, which focus primarily on potential hazards, and stability considerations, which address the impact of additional weight and equipment placement. For safety considerations, this subsection aims to identify the primary hazards associated with each technology without conducting an exhaustive risk assessment. Such an assessment would be necessary for a comprehensive assessment of the full range of risks associated with each technology.

The comparative analysis presented in Table 9 outlines the key features of each technology already discussed in Sect. 4 and in particular examines their impact on the safety and stability of the ship. In particular, cryogenic separation emerges as the most promising option, as it ensures safety and stability with a compact design and minimal impact on the ship's balance, while providing a bypass mechanism in the event of a system failure. This is closely followed by membrane technology, which requires flue gas pre-treatment and involves additional weight considerations. Chemical adsorption, on the other hand, involves significant plant weight and the use of hazardous solvents or by-products, but offers a bypass option. In contrast, pre-combustion and oxyfuel combustion are less important due to their potential impact on the safety and stability of the ship. These methods involve handling highly explosive H_2_ or concentrated O_2_ and there is no bypass option, so there is a risk of power failure on the ship in the event of a CC plant malfunction.

**Table 9:** Impact on safety and stability

CC method	Key features	Stability impact	Failure bypass	Risks for personnel	Potential to deal with the challenge
Cryogenic separation	Compact design minimises impact on ship stability; Possible bypass in case of failure; No added risks for personnel	Minimal impact	Yes	No	Highest
Membrane separation	Highly compact, flexible placement; Bypass possible in case of failure; Existing technologies may require extensive pre-treatment, adding weight	Less impact	Yes	No	High
Chemical absorption by NH3/MEA/PZ	Equipment size and weight have a negative impact on stability; Stability is influenced by the solvent present in both the absorber and the stripper; Solvent and/or by-products pose hazards; Bypass possible if failure occurs	Moderate impact	Yes	Yes	Moderate
Oxyfuel combustion	Increased hazard stemming from high concentrations of O_2_; Technical malfunctions can result in propulsion failure; No bypass option	High impact	No	Yes	Low
Pre-combustion	Significant risk arises from the generation of highly explosive H_2_; Technical defects may cause propulsion loss; No bypass possible; Ship's stability is adversely affected by the presence of heavy equipment and free surfaces	Significant impact	No	Yes	Lowest

The key features highlighted in Table 9 are based on an understanding of the respective technologies after reviewing the articles. All relevant references that support each technology’s characteristics are cited in Sect. 4, justifying the findings and aligning with the overview provided in Table 9.

#### Impact of ship movement and vibration

This section evaluates the impact of ship motion, as well as vibrations caused by machinery and weather conditions, on onboard CC technologies to identify the most suitable technology. Different CC methods vary in how they are affected by ship motion and vibration, as well as in their ability to overcome these challenges. Since these aspects were previously discussed in Sect. 4, repetition is avoided here. Table 10 shows the comparison, with membrane separation having less impact due to ship motion and vibration, and the highest potential due to its compact design and minimal number of moving parts. Cryogenic separation has a moderate impact due to its moving bed, but this can be easily mitigated, and it has high potential for overcoming this challenge. Chemical absorption has negligible impact due to ship motion, as shown in the study by Ros et al. [86], with moderate wear from moving parts, resulting in moderate potential. Oxy-fuel combustion has a negligible impact due to motion but can generate its own vibrations and cause moderate wear when integrated into a new engine. Pre-combustion is unaffected by ship motion but is significantly affected by ship vibration due to the heavy weight of moving parts, resulting in the lowest potential for overcoming the challenges of ship motion and vibration.

**Table 10:** Impacts by ship's movement and vibration

CC method	Key features	Ship's movement impact	Ship’s vibration impact	Potential to deal with the challenge
Membrane separation	Minimally affected by the motion of the ship; Only a few moving parts that could potentially be harmed by vibrations	Minimal impact	Minimal wear and tear	Highest
Cryogenic separation	Mobile bed might be influenced by the ship's motion, but this can be easily prevented; Vibrations could potentially escalate wear and tear	Moderate impact	Increased wear and tear	High
Chemical absorption by NH_3_/MEA/PZ	Flow of exhaust gas in the absorber column shifts when the ship is tilted, affecting the distribution of gases within the column; Despite continuous motion of the column, the capture rate tends to remain relatively constant	Negligible impact	Moderate wear and tear due to shifting parts	Moderate
Oxyfuel combustion	Unaffected by the ship's movement; System necessitates a new engine, producing its self-generated vibrations	Negligible impact	Moderate wear and tear; produces vibration	low
Pre-combustion	Syngas production remains stable despite the ship's motion, yet it is vulnerable to vibrations; Process is affected by vibrations due to numerous heavy-moving components, resulting in heightened wear and tear during adsorption	Negligible impact	Significant wear and tear due to heavy moving parts	Lowest

#### Engine load variation

The operating dynamics of marine engines differ considerably from those of industrial and power plants, as they primarily operate in an unstable state due to the fluctuating energy demand, which leads to fluctuations in the engine load. Fluctuating engine loads mean fluctuating exhaust gas temperatures, which emphasises the im-portance of developing heat exchangers that can cope with these temperature fluctuations for the optimal performance and lifetime of these technologies. Table 11 summarises the impact of different CC technologies on marine engines, focusing on addressing engine load variations as discussed in Sect. 4, while examining the advantages and disadvantages of each CC technology. Among the technologies, oxyfuel combustion stands out for its negligible impact, which positions it as the most promising to address this challenge. Chemical absorption, membrane separation and cryogenic separation have a moderate impact, which corresponds to a moderate potential to address this challenge. Conversely, pre-combustion has a significant impact due to the limitations of utilisation in steady-state operation and the need for additional equipment to provide energy during transient operation, so it has the lowest potential to address fluctuations in energy demand.

**Table 11:** Impact of engine load variation

CC method	Key features	Potential to deal with the challenge
Oxyfuel combustion	Capable to readily adapt to various loads by considering a buffer of O_2_; Adjustments to cooling energy are required for condensing water from exhaust gas	Highest
Chemical absorption by NH3/MEA/PZ	The diameter of the absorber is determined by the volume flow of exhaust fumes; The entire process must be designed to effectively capture CO_2_ at high loads	Moderate
Membrane separation &	No research discusses the effect of a varying CO_2_ mass flow on these technologies; Presumed to reliably capture CO_2_ at full load, resulting in an elevated energy demand	Moderate
Cryogenic separation		
Pre-combustion	Restricted to operating in a steady-state; Additional equipment is necessary to provide the energy required during unsteady operation modes; Utilising a dual-fuel engine in this context would lead to the emission of CO_2_	Lowest

#### Impurity tolerance

The presence of impurities in marine fuels, in particular sulphur emissions, is an important concern that is considered by the regulations of the International Maritime Organisation (IMO). The IMO's sulphur cap, which has been in force since 2020, limits the sulphur content in exhaust gases to minimise the environmental impact [126]. However, the regulation only limits the sulphur content in exhaust gases and not in the fuel itself. As sulphur-containing fuels are affordable, it is economically advantageous to install exhaust gas treatment systems on-fboard so that sulphur-containing fuels can continue to be used while complying with the regulations. This economic consideration also applies to the installation of on-board CC equipment, suggesting that CC technologies that can run on low-cost fuels would incur lower operational expenditure (OPEX).

Nevertheless, emissions of particulate matter (PM) and nitrogen oxides (NOx) from marine internal combustion engines (ICEs) pose a challenge for CC technology. Particulate matter from incomplete combustion can accumulate in CC plants, reducing efficiency and increasing costs. NOx emissions, which are regulated by IMO guidelines [127] and influenced by the choice of fuel, engine design and combustion temperature, are mitigated by techniques such as exhaust gas recirculation and cleaner fuels such as LNG, providing additional benefits for CC applications.

The tolerance of different CC technologies to contamination varies, as discussed in Sect. 4, which outlines the advantages and disadvantages of each CC technology. Table 12 provides an overview of how the different technologies included in the assessment are affected by impurities in both the fuel and the exhaust gases. In gas separation, cryogenic capture shows exceptional efficiency in dealing with impurities in the flue gas, as no degradation occurs in their presence. This is closely followed by chemical absorption with NH3/MEA/PZ, which occupies a remarkable and moderate position and requires pretreatment to avoid solvent degradation. Pre-combustion is moderately affected and requires additional pre-treatment to avoid non-convertible impurities. Oxyfuel combustion is less important in this respect. It requires clean fuel and a post-treatment system. Membrane separation is the least important. It is characterised by a negligible tolerance to exhaust gas impurities and requires extensive pre-treatment to prevent membrane damage.

**Table 12:** Impact of impurities in exhaust

CC method	Key features	Potential to deal with the challenge
Cryogenic separation	There is no deterioration caused by impurities in exhaust gases; Core process not affected by impurities	Highest
Chemical Absorption by NH3/MEA/PZ	Pre-treatment is essential to prevent the degradation of the solvent, while sulphur from exhaust gases can be converted into a valuable by-product, such as ammonia, if desired	High
Pre-combustion	Additional pre-treatment is required to avoid non-convertible impurities; Absence of O_2_ in syngas prevents degradation; Wide variety of fuels can be utilised with additional treatment	Moderate
Oxyfuel combustion	Clean fuel is necessary; Any contaminants must be addressed by an additional aftertreatment system	Low
Membrane separation	Extensive pre-treatment needed to avoid membrane damage; Impurities in exhaust gases have a significant impact, with water in exhaust fumes causing degradation of membranes	Lowest

The references supporting these statements are provided in Sect. 4, where the details of each technology and their interaction with impurities are discussed, and all relevant sources are cited accordingly.

In addition to handling impurities during the CC process, it is crucial that the captured CO_2_ meets the purity standards required for downstream storage or utilisation. Impurities in the captured CO_2,_ such as sulphur compounds, nitrogen oxides, or particulate matter, can affect the integrity of storage reservoirs and reduce the effectiveness of CO_2_ utilisation processes, such as enhanced oil recovery or chemical synthesis. Therefore, CC technologies must not only tolerate impurities in the exhaust gas but also incorporate adequate purification steps to ensure the captured CO_2_ complies with these downstream requirements. This aspect further emphasises the importance of pre-treatment and post-treatment systems in achieving both compliance and operational efficiency.

#### Discussion on the initial stage assessment

To identify the promising on-board CC technologies that have successfully passed the initial phase, a summary table (see Table 13) is provided to assess their potential in overcoming the five challenges described in this section. Solid adsorption, which is not considered compatible with ICEs due to its lower adsorption capacity at high temperatures, is excluded from further analysis.

**Table 13:** Summary table comparing the potential of each CC technology

CC method	Potential to tackle challenges					
	ICE compatibility	Ship’s safety and stability	Ship’s movement and vibration	Fluctuations in energy demand	Impurity tolerance	Selected CC technology
Pre-combustion	Yes	Lowest	Lowest	Lowest	Moderate	No
Oxy-fuel combustion	Yes	Low	Low	Highest	Low	No
Chemical absorption by NH3/MEA/PZ	Yes	Moderate	Moderate	Moderate	High	Yes
Solid adsorption	No	←––––––Not considered further––––––→				No
Membrane separation	Yes	High	Highest	Moderate	Lowest	Yes
Cryogenic separation	Yes	Highest	High	Moderate	Highest	Yes

As for pre-combustion technology, it shows minimal potential when it comes to ship safety, stability, motion, vibration and fluctuations in energy demand. Therefore, this CC technology is excluded from further comparative analysis. While oxyfuel combustion shows the greatest potential in coping with fluctuations in energy demand, it performs poorly in the other three challenges, so it is also excluded.

Among the remaining three CC technologies, cryogenic capture shows the most balanced potential, with two highest, one high and one moderate potential, making it the most deserving candidate for the next stage of the analysis. This is closely followed by membrane technology with one highest, one high, one moderate and one lowest potential. Although it has the lowest potential in removing exhaust pollution, the implementation of successive pre-treatments could mitigate this challenge, albeit with an additional energy input. The third candidate to move up to the next stage of the comparison is chemical absorption, which has one high and three moderate potentials to overcome the challenges mentioned.

### Follow-up assessment

At this stage, the authors consider the three most prominent CC technology found in initial stage assessment, namely: Cryogenic separation, Membrane separation, and Chemical absorption technology for further consideration. The challenges listed at this stage include:

- The current level of development for the technology, and its anticipated readiness for commercial on-board operation.
- Space utilisation for CC plant installation, including associated CO_2_ storage tanks, and its impact on the cargo capacity of a ship.
- Increase in energy demand due to on-board generation of all the energy required to operate the CC plant.
- Level of CO_2_ capture rate, considering the associated energy penalties
- Added weight due to the installation of a CC plant, along with the intermediate storage of captured CO_2_ and necessary chemicals.
- Initial investment costs and operational expenses, particularly the cost-effectiveness of capturing one ton of CO_2_.

#### Maturity level

To assess the maturity of CC technologies for onboard installation, this study aims to evaluate the Technology Readiness Levels (TRLs) of these technologies through literature reviews considering (a) Onboard CC usage and (b) Commercial usage in general industries. Typically, TRLs are categorised into three phases: early stage (TRL 1–3) for pure research, development phase (TRL 4–6) for scaling up and validating technology, and demonstration phase (TRL 7–9) for transitioning from pilot to commercial service [128]. The assessment of maturity level is based on these three categories.

In Sect. 4, 26 reports/papers describing the onboard usage of CC by chemical absorption are reviewed. Among these, only one paper on the CC-Ocean project [35] explains the operation of a demo plant by the ship’s crew, assigning a TRL 7 level for it for onboard operation. The other listed reports/papers primarily involves simulation-based studies and case studies, which might fall within TRL 2 and TRL 3. On the other hand, considering commercially available post-combustion CC plants, the most common and successfully used amine solvent for chemical absorption is 30 wt% MEA [89], considered successful at TRL 9. NH_3_ absorption technology is evaluated at TRL 6 based on successful testing in pilot plants [83]. Concentrated piperazine (PZ) and its absorbent capabilities achieve TRL 6, with CO_2_ capture rates of 83.1–99.1% [84] Ionic liquids for CC are in early research stages, with a TRL of 2–3 based on laboratory tests [85].

Regarding membrane technology, Sect. 4 identifies only two papers which consider literature reviews and simulation studies for onboard application; therefore, the TRL level is identified as 2. On the other hand, MGS technologies for CO_2_ removal from natural gas are widely used in industrial settings. However, applying them to flue gas separation faces challenges due to the large volume of feed gas with low CO_2_ concentrations [98]. This results in high operational costs, limiting their adoption in large-scale applications. Despite their potential, membrane separation technologies are currently assessed at TRL 5, primarily used in research and development stages, including pilot plants.

For Cryogenic separation, only two studies were identified in Sect. 4. One of those performed a case study on a car carrier and a RoPax ferry, evaluating CO_2_ reduction, ship stability, and economic factors while using advanced cryogenic CC (A3C) process. The other paper explored conceptual designs for an onboard CC system aiming to store captured CO_2_ as dry ice. Therefore, a TRL level of 2 is assigned to both of it. On the other hand, Cryogenic separation technology, tested in pilot plants at various scales, achieved over 90% CO_2_ capture rates under real flue gas conditions [129]. Small-scale CFG pilot plants ran successfully for several weeks, reaching capture rates of up to 95%. Although cryogenic gas separation is already commercially implemented in the industry, the process is not yet fully developed. According to these findings, cryogenic separation technology is currently at a TRL of 4. However, considering the maturity of technology in the industry, cryogenic separation has the potential to soon reach a higher TRL.

The comparison result is summarised in Table 14. A higher TRL increases the likelihood of a commercial application in the near future. As TRL rises, cost estimates become more precise due to the accumulated experience and understanding of these technologies. The table shows that post-combustion by chemical absorption is the most promising, having already achieved TRL 7 for onboard applications. In contrast, membrane separation and cryogenic separation show high and intermediate potential, respectively for onboard implementation. Limited research has been conducted on the onboard application of the latter two technologies, resulting in a low TRL. However, considering their potential usage in commercial pilot plants, the authors anticipate both having the capacity to achieve higher TRLs for onboard applications very soon.

**Table 14:** Maturity level

CC method	TRL (for commercial application)	TRL (at most for onboard application based on reports/papers)	Potential to deal with the challenge
Chemical absorption by NH3/MEA/PZ	MEA: 9; NH3: 6; PZ: 6	7	Highest
Membrane separation	5	2	High
Cryogenic separation	4	2	Low

#### Space constraints

The constrained space presents a considerable obstacle to installing CC equipment on ships. The area taken up by the CC plant and associated installations cannot be utilised for transporting goods, which impacts the ship's primary function and economic viability. As the Energy Efficiency Design Index (EEDI) considers transported cargo in its calculation, reduced cargo capacity negatively impacts the attained EEDI. Therefore, a CC unit designed for onboard ship applications should aim to minimise space requirements.

In the context of CC via chemical absorption, the size of absorber and stripper units differs depending on the targeted CC rate from flue gases. Achieving a higher fraction of captured CO_2_ necessitates taller absorber columns to accommodate an extended mass transfer zone, while the column diameter determines the maximum flow rates, leading to larger spatial requirements. Moreover, to minimize the energy needed for solvent regeneration, a bulky lean-rich heat exchanger is essential. For MEA processes, the dimensions of absorber, stripper, and associated components contribute to significant space requirements and weight, especially in large-scale applications [45]. As onboard applications face challenges due to space constraints, rotating packed-beds show promise for reduced unit sizes in this regard [130]. Increasing solvent flow rate in the reboiler can reduce absorber and stripper size, but it raises energy consumption and OPEX. While a lower height of absorber and stripper reduces CAPEX and space demand, it increases OPEX [87]. Overall, space requirements in absorption technologies depend on the CC application, with higher mass flows and velocities requiring larger components. The authors approximate that the space requirements for the compared absorption processes are comparable, given the general similarity in setup across alternative solvents. However, among the three technologies evaluated in the assessment, the chemical absorption process necessitates the most space.

In membrane technology, the low CO_2_ concentrations in flue gas often require multiple membrane units or larger membrane contact surfaces, thereby increasing the space needed for the CC [101]. The space occupied by membranes also varies with the flow rate of the flue gas, with higher flow rates necessitating larger CC plants. Additionally, compressor units, which are essential for the operation of membrane gas separation (MGS) technology, contribute to additional space requirements. The extensive pre-treatment of flue gases before CO_2_ capture is another significant factor contributing to the space requirements of this technology [98]. But membrane technologies are designed for applications with limited space, and currently, they are utilised on ships for tasks such as freshwater production and wastewater treatment [131]. The unique structure of membranes enables a large surface area for separation within confined spaces. Similar to existing membrane applications onboard, it is expected that the required pre-treatment may occupy more space than the membranes themselves. Nevertheless, the adoption of cleaner fuels has the potential to decrease the required pre-treatment equipment, thus likely reducing space requirements. The authors assess that the CC process using membranes is expected to occupy less space compared to chemical absorption technologies.

Heat exchangers play a significant role in determining the space requirements of cryogenic separation technology, with a variety of types including tubular, coil, or plate heat exchangers commonly utilised [75]. There may be a requirement for multiple heat exchanger units, especially for managing large volumes or high velocities of flue gas, which can result in heightened space demands. However, the dimensions of the cryogenic separation unit only undergo minor changes when capturing a higher CO_2_ flow. Willson et al. (2020) proposed cryogenic separation setup is characterised by a compact unit, contrasting with the multiple bulky columns needed for the absorption process. The authors, however, posit that cryogenic separation likely requires less space than chemical absorption but more than membrane separation.

Table 15 shows the comparison of the three CC technology where membrane separation ranks the highest, followed by cryogenic separation and chemical absorption, respectively.

**Table 15:** Space utilisation

CC method	Key features	Potential to deal with the challenge
Membrane separation	Designed for use in limited spaces	Highest
Cryogenic separation	High flue gas volumes or velocities may require multiple heat exchangers, increasing space needs; Designed with minimal variations in size, even with increased CO_2_ flow	Moderate
Chemical absorption by NH3/MEA/PZ	System consists of several bulky elements	Low

#### Onboard energy utilisation

The required energy for the onboard CC plant must be generated onboard which requires additional fuel to burn. Increased energy demand from the CC unit leads to higher CO_2_ emissions due to escalated fuel consumption, consequently diminishing carbon reduction efficiency. As fuel consumption is a key operational cost for ships, this will directly influencing the operational cost (OPEX) as well.

Regarding post-combustion by chemical absorption, one of the biggest advantages is utilising engine waste heat for solvent regeneration. According to the study conducted by Awoyomi et al. [46], the reboiler duty accounts for only 27% of the energy needed for regenerating MEA solvents. In addition, NH_3_ solvents allow CO_2_ desorption at elevated pressure, reducing energy needs for compression.

On the other hand, in membrane separation, high partial pressure difference of CO_2_ is crucial, necessitating energy for compression or vacuum creation. While membrane separation has low-energy demand, pre-treatment substantially reduces its energy efficiency. MC membranes enable the utilization of waste heat onboard for solvent regeneration. Among the technologies under consideration, membrane separation is expected to have the lowest energy demand when pre-treatment is not taken into account. However, the energy required for the necessary pre-treatment significantly diminishes its energy efficiency.

For the cryogenic separation, it relies on extremely low-temperatures (− 100 °C) to solidify CO_2_ in flue gases for separation, demanding significant power for the refrigeration unit. Apart from that, it cannot utilise the waste heat from flue gas. A case study conducted by Willson [108] estimated the energy demand for capturing 3.7 t_CO2_/h to be 1700 kW, with OPEX projected to be 70% lower than the benchmark absorption process with MEA.

Table 16 presents a comparison of three CC technologies. Chemical absorption is rated highest because of its ability to use waste heat for solvent regeneration and desorption at elevated pressure. Following closely is membrane separation, but it necessitates energy for substantial pre-treatment, with limited waste heat utilisation. Despite membrane separation's overall energy demand being comparable to chemical absorption, there is a lack of reports/papers providing figures for verification. Cryogenic separation ranks last due to its inability to use waste heat and its substantial electrical energy requirement for the refrigeration process.

**Table 16:** Onboard energy utilisation

CC method	Key features	Potential to deal with the challenge
Chemical absorption by NH_3_/MEA/PZ	Utilisation of waste heat is possible; Desorbing at high pressure conserves energy in CO_2_ compression	High
Membrane separation	Requires additional energy for intensive pre-treatment and pressure creation; Limited waste heat usability	Moderate
Cryogenic separation	Unable to use waste heat; requires substantial electric energy for the refrigeration unit	Low

#### Capture rate

In general, CC technologies can achieve a capture rate of 99%, but the reports/papers suggest that this is not economically feasible [132]. The high costs associated with capturing the last 10% of CO_2_ have led most research to focus on achieving a 90% capture rate. The main cost increase for complete capture systems results from the need for larger plants to capture CO_2_ at very low partial pressure. Each technology has different technical and energy requirements to achieve higher capture rates.

Chemical absorption requires a considerable column height to increase capture rates. Improving CO_2_ capture requires minimising the solvent concentration in the upper part of the absorption column. This requires a higher regeneration rate, resulting in a higher load on the reboiler and a larger stripper column.

On the other hand, membrane separation is based on a pressure difference [98], whereby the energy requirement increases with decreasing CO_2_ concentration. In addition, the material thickness of the membrane must be improved to withstand higher pressures. Furthermore, beyond a certain pressure threshold, there is a risk of other flue gas components penetrating the membrane.

Cryogenic separation relies on extremely low-temperatures to solidify CO_2_. The low-temperature of the gas stream reduces the solubility of CO_2_ and thus enables a higher capture rate with less effort than alternative technologies. The use of an elevated moving bed of metal beads in the advanced cryogenic capture process (A3C) is seen by Willson [108] as a practical strategy to achieve a capture rate of 99%.

Given the variability in experimental setups across studies (e.g. different operating conditions, fuel types, and engine configurations), direct quantitative comparisons of capture rates would be misleading without a uniform baseline. This is why Table 17 shows a qualitative comparison of three CC technologies considering the CC rate. Cryogenic capture has a high potential as it can capture 99% of CO_2_ with less effort than alternatives. This is followed by chemical absorption, which requires a higher absorber and stripper with a higher power consumption for the regeneration process to increase the CC rate. The last option is membrane capture, which requires high pressure to achieve better capture, but the strength of the membranes is not sufficient to withstand this high pressure.

**Table 17:** Capture rate

CC method	Key features	Potential to deal with the challenge
Cryogenic separation	Reduced temperatures result in lower solubility in the flue gas stream; A raised bed of metal beads can capture 99% of the CO_2_ present in the flue gas stream	High
Chemical absorption	Significantly larger absorber and stripper units are required, resulting in a notable increase in power consumption for the regeneration process	Moderate
Membrane separation	Achieving a better capture rate necessitates higher pressure; Insufficient material to withstand the increased pressure	Low

#### Additional weights

Retrofitting an existing ship with a CC system will result in a reduction in DWT due to the additional weight, which will affect the capacity of the ship, while for new designs a modified hull design can accommodate the additional weight.

Chemical absorption includes various components that increase with increasing volumetric flow and decreasing CO_2_ concentration in the exhaust gases. Feenstra et al. [45] estimated the weight of CC equipment for a 3000 kW cargo ship adding 80 tonnes to the ship's weight. However, taking into account the weight of the CO2 storage tank, the temporarily stored CO_2_ and the LNG consumption for maximum CO_2_ storage, the total additional weight was estimated at 420 tonnes.

On the other hand, the weight of the cryogenic separation plant is influenced by the amount of flue gas treated, especially by the heat exchangers, the cooling units and the bed of moving metal balls. Despite the compact design, the equipment required is heavy, estimated at 100 tonnes for cases where only the main engine exhaust is treated, according to Willson [108]. In addition, the CO_2_ storage tanks filled with liquid CO_2_ represent the heaviest additional load on the ship. The weights for chemical absorption and cryogenic capture are based on different case studies and are therefore not harmonised for comparison.

Weight data for membrane separation was not available, but it is assumed to be lighter than other technologies. It is assumed that the weight of the membrane plant, even with pre-treatment equipment, is lower than for cryogenic separation. The use of clean fuels further reduces the weight of the plant by minimising the amount of pre-treatment required.

Table 18 shows a comparison of the three CC technologies, taking into account the additional weight of the ship. Membrane separation harbours a high potential for reducing the weight of the ship. Both cryogenic separation and chemical absorption have a moderate potential, as chemical absorption requires heavy components and cryogenic separation requires additional cooling units.

**Table 18:** Additional weights

CC method	Key features	Potential to deal with the challenge
Membrane separation	Its weight assumed lower than other technologies; Cleaner fuels reduce equipment weight	High
Cryogenic separation	Weight correlates with the CO_2_ flow rate; Requires refrigeration units	Moderate
Chemical absorption	Weight is corresponding to the CO_2_ flow and CO_2_ concentration in flue gas; Several heavy-weighted components are required	Moderate

Given the variability in the data from different case studies, each with unique operational conditions and system configurations, a qualitative assessment provides a more reliable comparison of the technologies' impact on ship weight. Direct quantitative comparisons could be misleading due to differences in the setup of each study. Therefore, the approach used in Table 18 is best suited for understanding the relative weight impacts of these carbon capture systems.

#### Cost implications

The cost efficiency of CC technologies is influenced by various factors. A lower concentration of CO_2_ in the flue gas and a reduced quantity of flue gas result in higher costs per captured tonne of CO_2_, showcasing an economies-of-scale effect. Conversely, a higher capture rate significantly lowers costs [45]. Regarding cost implications, this study considers the capital expenditure (CAPEX) and operational expenditure (OPEX) of each of these three CC technologies to evaluate the overall impression for onboard installation.

The deployment of chemical absorption equipment in power plants involves significant CAPEX, primarily due to absorber and stripper unit costs, with packed columns being major contributors [133]. However, it presents trade-offs between CAPEX and OPEX [87]. Decreasing the size of the installed absorber reduces initial costs but requires a higher recirculation rate of the solvent and increased reboiler duty. These adjustments result in elevated power consumption, constituting the primary contributor to OPEX. The selected solvent for the absorption processes can alter the CAPEX and OPEX. While different solvents may have similar CAPEX, OPEX varies. MEA has a low purchase price but demands significant energy for regeneration, leading to increased OPEX [77]. K_2_CO_3_ has a lower purchase price, reduced solvent needs, and lower regeneration energy demand compared to MEA [133]. NH_3_ requires less energy for absorption than MEA, resulting in lower OPEX [46]. PZ and K_2_CO_3_ also have lower OPEX due to their resistance to solvent degradation, requiring less replenishment [77]. It means, alternative solvents such as K_2_CO_3_, NH_3_, and PZ can significantly reduce OPEX compared to MEA, attributed to lower purchase prices, reduced energy demands, and longer service lifetimes. NH_3_ stands out with the least OPEX, while PZ demonstrates about 15% lower energy demand for solvent regeneration compared to MEA, translating to 85% of the benchmark process's OPEX [134]. Awoyomi et al. [46] designed an absorption unit with aqueous-ammonia solvent for a 10,305 kW LNG-fuelled engine. The estimated CAPEX is around $35 million, covering CO_2_ compression and liquefaction. Notably, storage tank costs were excluded in the simulation, which focused on CC onboard a CO_2_ tanker.

Regarding membrane separation, its cost remains uncertain as no figures were available in the reports/papers. However, producing these required membranes is known to be challenging and likely expensive due to their high-tech nature. The present membrane technology necessitates extensive pre-treatment, contributing to the overall investment costs. Additionally, the low concentration of CO_2_ in the exhaust gases of internal combustion engines suggests that multiple membrane units may be needed [98] and [100], further contributing to higher costs. In general, membrane technology is regarded as having the highest investment costs among the three technologies. Comparable to CAPEX, there are no available figures for OPEX in membrane separation in the consulted reports/papers. While energy requirements for the separation process are anticipated to be low, potentially even lower than obligatory pre-treatment, maintenance costs emerge as the primary cost driver for membrane technology. The short lifespan of currently available membranes, coupled with their high expense, leads to membrane technology being evaluated with the highest OPEX compared to other technologies in this assessment.

Compared to MEA absorption technologies for CC, cryogenic separation technology offers substantial potential for cost savings [107]. When integrated into the design of a new-build power plant, the CAPEX of a cryogenic separation plant is only half of the costs associated with an amine-based absorption process. This also applies to the energy penalty, as cryogenic separation technology requires only half of the respective load of an MEA plant [104] and [107]. Willson's [108] case study on the A3C process for a 12,614 kW LNG-fuelled engine estimates a CAPEX of £11.5 million, ranking it highest due to significantly lower costs compared to absorption plants for less powerful engines.

In contrast to other technologies, cryogenic separation solely requires electricity to operate the cryogenic processes, eliminating the need for excessive heat energy for solvent regeneration. Consequently, the power demand of cryogenic separation technology is lower, resulting in reduced fuel costs in OPEX compared to other technologies. Willson also estimates an OPEX of about £1.15 million for his case study, with fuel costs for energy generation contributing approximately 80% to the overall OPEX. The A3C process is recognized for its potential to reduce OPEX by 70% compared to conventional industrial processes.

Based on the above discussion, comparison Table 19 is prepared for these three CC-considering cost implications while onboard installation. Here, Cryogenic separation demonstrates a moderate CAPEX and low OPEX, positioning it as a high potential to minimise the cost implication. On the other hand, chemical absorption comes with high CAPEX and moderate OPEX, suggesting a moderate potential to reduce the expenses. In contrast, membrane separation exhibits high CAPEX, the highest OPEX, and a comparatively lower potential to minimise the cost implications.

**Table 19:** Cost implications

CC method	Key features	CAPEX	OPEX	Potential to deal with the challenge
Cryogenic separation	CAPEX is about half of what required for amine-based absorption process; About 80% of total OPEX originate from increased energy demand; Exhibits 70% lower OPEX compared to conventional industrial processes	Moderate	Low	High
Chemical absorption	Significant initial investment costs; Balancing CAPEX and OPEX allows for cost reduction by downsising the plant; Low chemical costs if proper solvent is selected	High	Moderate	Moderate
Membrane separation	Potential for high costs due to advanced technology and pre-treatment requirements; Core process has low-energy demand; Primary cost contributor is significant maintenance expenses, attributed to the short lifespan of membranes	High	Highest	Low

Due to gaps in cost data, a qualitative approach is the best option for comparing these technologies. While a quantitative comparison would be clearer, insufficient empirical data, particularly for membrane separation and cryogenic systems, limits its use. Qualitative analysis offers a more flexible evaluation, considering existing case studies, technological maturity, and operational factors. This method accounts for emerging trends, such as economies-of-scale in cryogenic separation and innovations in membrane technology, making it ideal when direct cost data are lacking or inconsistent.

#### Discussion on the follow-up assessment

To identify promising onboard CC technologies and assess their potential in addressing the six challenges outlined in this section for onboard installation, a summary table, Table 20 is constructed for comparison.

**Table 20:** Comparison of the potential of three CC technologies passing the initial stage for the follow up assessment

CC method	Potential to tackle challenges						
	Maturity level	Space constraints	Onboard energy utilisation	Capture rate	Additional weights	Cost implications	Selected CC technology
Chemical absorption	Highest	Low	High	Moderate	Moderate	Moderate	Most favourable for ships that can accommodate larger CC technology equipment
Cryogenic separation	Moderate	Moderate	Low	High	Moderate	High	Favourable for confined space, but extra energy is needed
Membrane separation	High	Highest	Moderate	Low	High	Low	Favourable for confined space, but expensive

Of the three CC technologies, chemical absorption is characterised by its highest degree of maturity, which makes it a promising solution for CC on-board. Despite its high potential in addressing the energy demand challenge, it shows only moderate potential for capture rate, which adds weight and cost. The method also reaches its limits when it comes to space requirements. However, the potential, which is categorised as moderate, can still be improved to achieve a higher potential. Considering this fact, chemical absorption only struggles with space issues, which could make it suitable for newly built or retrofitted ships that can accommodate the larger CC technology equipment. However, the choice of CC technology depends on factors such as engine power, space on-board and economic considerations. Therefore, CC by chemical absorption may have limited suitability for ships due to space requirements, potential reduction in cargo capacity and negative impact on the Energy Efficiency Design Index (EEDI).

On the other hand, cryogenic capture with a moderate level of maturity has a low potential for utilising waste heat to cover energy needs. Nevertheless, the technology shows a high potential for reducing overall costs and capturing more carbon. In terms of space utilisation and additional weight, it has moderate potential. The challenge lies in the additional electrical energy required for the cooling units. However, this technology could prove to be more advantageous in confined spaces, despite the higher energy requirement during operation.

Membrane separation, on the other hand, has a high potential with its mature technology and the highest capacity for space utilisation due to its compact design. The total weight of the entire CC components is also lower compared to the other two technologies. However, the technology is expensive due to the additional pre-treatment requirements and the cost of the membranes, combined with a low potential for CC rate. Similar to cryogenic capture, this technology may find more favourable applications in confined spaces despite its higher overall cost.

## Conclusions

This paper provides an in-depth review of CC technologies and analyses their process flows, advantages, disadvantages, and recent advances through a literature review. A particular focus is placed on assessing the suitability of these technologies for use on-board ships, considering the particular challenges posed by the shipboard environment. A comprehensive comparative assessment is conducted, ana-lysing each technology based on factors such as economic feasibility, capture rates, maturity, energy requirements, space requirements and other relevant considerations. The main conclusions from this study are as follows:

- CC serves as a transitional solution, paired with fossil fuels, until complete reliance on alternative fuels is feasible.
- Six CC technologies—pre-combustion, oxyfuel combustion, post-combustion by chemical absorption, adsorption by solid sorbents, membrane separation and cryo-genic separation—are discussed in detail with regard to their operating principles, advantages and disadvantages as well as their potential on-board applications.
- Chemical absorption technology lends itself to commercial implementation supported by a mature level and extensive literature, while adsorption technology is considered impractical for ICEs and on-board applications due to issues such as temperature sensitivity, oxidation of amine adsorbents and incompatibility with sulphur and nitrogen impurities.
- Storing CO_2_ in gaseous form is often considered impractical due to its significant volume, despite the advantages of lower pressure and the requirement for refrigeration. However, the feasibility of gaseous CO_2_ storage depends on various factors, such as operational profiles, ship arrangements and associated costs and energy requirements. While the supercritical liquid phase is favoured for pipeline transport, CO_2_ storage in solid form on-board ships is promising but still in the development stage. Opting for storage in liquid form, in particular maintaining a pressure of 15 bar at − 27 °C, is considered an optimal choice that offers advantages in pump handling and contributes to lower life cycle costs (LCC) and net present value (NPV) for retrofitted ships.
- A total of 11 challenges for the implementation of CC technologies on-board have been identified, leading to a comparative evaluation of these technologies to assess their potential to overcome these hurdles.
- In the initial phase, the CC technologies are evaluated against five challenges: ICE compatibility, ship safety and stability, ship motion and vibration, engine load variations and tolerance to contaminants in exhaust gases to determine their feasibility for on-board application. The top three CC technologies—post-combustion by chemical absorption, membrane capture and cryogenic capture—were identified as having enough potential to proceed to the next stage of evaluation, where they must overcome six remaining challenges: Maturity, space requirements, on-board energy utilisation, CC rate, additional weight and cost implications.
- The chemical absorption process proves to be the most promising process for use on-board ships. It is suitable for both new builds and retrofits, especially if they can accommodate the large space requirements of the process equipment.
- For ships with limited machinery space, membrane and cryogenic separation processes are considered suitable options, with a crucial trade-off between cost and energy requirements. Membrane separation is more expensive, while cryogenic separation requires more energy.

The study recognises that there is a lack of comparable data in the reports/papers during its comparative analysis. To address this, it is important to create a common basis that considers factors such as available space and energy. In addition, conducting case-specific assessments is crucial to determine the most effective CCS technology for applications on-board ship.

## Data Availability

The authors declare that no specific data have been created or used in this review article submission.

## Appendix

See Tables 21, 22, 23, 24, 25, 26.

**Table 21:** Overview of single chemical solvents used in CC

Absorbent	Advantages	Disadvantages	Energy demand for regeneration (MJ/kgCO2)	Absorption rate (relative to MEA)	Effectiveness for using onboard	Remarks	Reports/papers
Monoethanolamine (MEA)	High Absorption Capacity, proven Technology, Low costs	Energy Intensive, Corrosive nature, Easy to degrade, volatile	2.2–6	1	30 wt% MEA is widely studied and effective choice in diverse ship types [44, 99, 101]	8.2–14% efficiency penalty for a power plant	[66, 77, 82, 133]
Piperazine (PZ)	Less energy-intensive than MEA, Good thermal stability, Resistant to corrosion	Causticity and toxicity	About 85% of MEA	2	30 wt% ME is identified as better solvent than 30–40 wt% PZ [100], whereas MEA/PZ is preferred against MEA [98]	Can be used as a blend to MEA to increase efficiency (activated MEA)	[45, 66, 77, 81, 134]
Aqueous ammonia	Wide range of sources, lower chemical costs than MEA, resistant to thermal and chemical degradation	High volatility	Very low, requires only 27% reboiler duty compared to MEA	0.05–0.33	30 wt% MEA perform better when comparing to 2–28 wt% aqueous ammonia [100]	Tested in pilot-scale only	[46, 66, 77, 135]
Ionic liquids	Less costs than MEA; resistant to thermal and chemical degradation, non-volatile	Particularly toxic to aquatic organisms	Lower than MEA	Comparable to or higher than MEA	Early research stages	Some liquids show high regeneration efficiency (up to 95%), tested in pilot-scale only	[77, 138, 139]
Potassium carbonate (K_2_CO3)	High thermal & chemical stability, non-volatile, no degradation, more efficient than MEA	Low mass transfer rate, higher space demand than MEA	Lower than MEA	NA	K_2_CO3 is useful in pressurized combustion and fuel reforming systems [124]		[77, 82, 133]

**Table 22:** Reports/papers on post-combustion chemical absorption for the application onboard ships

Authors (year)	Aim of study	Engine type	Ship type	Solvent	Key findings	Additional information
Zhou and Wang [136]	CC and storage—Solidification and storage of carbon dioxide captured on ships	NaOH, CaO	Not specified		A novel method for solidifying CO_2_ captured from marine engine exhaust for economical onboard storage on ships. Operational costs of the proposed method are evaluated and compared with traditional CO_2_ liquefaction, highlighting its potential as a cost-effective solution for mitigating emissions from marine sources	Profit from selling product of CC is able to exceed operational costs
Luo and Wang [44]	Study of solvent-based CC for cargo ships through process modelling and simulation	Two four-stroke reciprocating diesel engines at a total power of 17 MW	General cargo ship	MEA	Two integration options were simulated to analyse thermal performance and estimate equipment size for combining the ship energy system with the CC process. Results show that CC can only reach 73% due to limited heat and electricity supply for CCS in the integrated system	By adding a supplementary gas turbine to boost energy supply for the capture plant, the CC level could reach 90%. However, the cost of captured CO_2_ is approximately 163.07 €/ton CO_2_, primarily due to a 21.41% increase in fuel consumption for the additional diesel gas turbine
Van Den Akker [116]	CC onboard LNG-fuelled vessels: A feasibility study	8000 DWT, 3000 kW dual-fuel LNG is used	General cargo	30 wt% MEA	An emission reduction of 87% is found compared to the reference ship. The gross cost per ton of CO_2_ avoided is approximately €74, with net costs being less than €20. While slightly higher than land-based CC projects, it remains in the same order of magnitude	Establishing a price for CO_2_ emissions could make onboard CC profitable, with a €20 per ton CO_2_ emitted making it cost neutral
Wang et al. [65]	Reviews on current carbon emission reduction technologies and projects and their feasibilities on ships	6300 TEU, 57,059 kW	Container ship	N/A	High energy, material and space requirements as constraints, further development necessary	A concise overview of the current emissions landscape, policies, and technologies. Predictions for future developments and a comparison of various CC technologies. Includes examples of current incentives for CCS
Feenstra et al. [45]	Study of ship-based CC onboard of diesel or LNG-fuelled ships	Two reference ship engines of 1280 KW and 3000 kW were chosen (Diesel or LNG)	Inland ship 1280 KW, General cargo ship 8000 DWT, 3000 kW	30 wt% MEA and 30 wt% PZ	Integrating the thermal energy of exhaust gas with the stripper reboiler resulted in reduced CAPEX and OPEX for diesel and LNG-powered ships. In LNG ship, the evaporation of LNG's cooling capacity was utilised to liquefy the captured CO_2_	Diesel options are more expensive due to additional refrigeration unit for liquefying CO_2_
Fang et al. [140]	Optimal Sizing of Shipboard CC System for Maritime Greenhouse Emission Control	Not specified	All-electric ship	N/A	Deploying Energy Storage Systems (ESS) alone cannot meet more stringent GHG emission control targets in the future. However, with the combined deployment of CCS and ESS, GHG emission reductions ranging from 10 to 60% can be achieved	The corresponding average CC level increases 11.9% with the joint management
Balcombe et al. [24]	How to decarbonise international shipping: Options for fuels, technologies and policies	Not specified	Not specified	N/A	A comprehensive approach involving a mix of fuels, technology, and policy, along with short-term incentives, is essential for the maritime sector's rapid and equitable decarbonization	A summary of currently studied technologies and policies designed to mitigate shipping emissions
Awoyomi et al. [119]	CO_2_/SO_2_ emission reduction in CO_2_ shipping infrastructure	10,200 kW HFO is used	CO_2_ carrier	NH_3_	At 85% load with the CCS system in operation, the maximum recovered heat is 4MWth, enabling the capture of 70% of CO_2_ and 98% of SO_2_	NH_3_ as a solvent enables the simultaneous removal of SO_x_ and CO_2_, with the potential for selling the by-products, simulations cover various ship operation modes and are based on the Munmorah pilot plant for the CC process
Awoyomi et al. [46]	Process and Economic Evaluation of an On-board Capture System for LNG-Fuelled CO_2_ Carriers	10,305 kW dual-fuel LNG is used	CO_2_ carrier,	NH_3_	The design of the CC system must consider the operational profile specific to the ship	Exhaust Gas Recirculation (EGR) diminishes plant size and enhances capture efficiency, with the lowest achieved cost being 117 $/tCO_2_ at a capture rate of 90%
Lee et al. [122]	Comparative analysis of on-board methane and methanol reforming systems combined with HT-PEM Fuel cell and CO_2_ capture/liquefaction system for hydrogen fuelled ship application	3000 DWT, 3800 kW Methane and methanol	General cargo	MEA	Excess heat from HT-PEMFC and reformer in both methane- and methanol-based systems supports CO_2_ capture, with LNG cold energy reducing compressor power consumption in the methane system. Despite slightly higher compressor power in the methanol system, it maintains higher efficiency	Conducting an energetic analysis of methane/methanol-based systems for comparison
Long et al. [88]	Improvement of marine CC onboard diesel fuelled ships	3000 kW diesel engine	Not specified	MEA, MEA/PZ, MDEA/PZ	MEA/PZ and MDEA/PZ show respective increases of 1.7% and 2.8% in CO_2_ removal compared to the MEA solution. The MDEA/PZ process with an intercooler achieves 90.6% CO_2_ removal, while the MDEA/PZ process with multiple feeds achieves 90.5%	Several mixed solvents, an intercooler and multiple feeds are used to improve CO_2_ removal in the absorber
Lee et al. [137]	Novel methodology for EEDI calculation considering onboard CC and storage system	3800 TEU, 53,000 DWT, 18,200 kW with dual-fuel LNG with pilot fuel is used	Container feeder	22 wt% MDEA with 8 wt% PZ	The CO_2_ capture rate must exceed the planned EEDI reduction to offset the additional power demand for CC	The engine's waste heat is enough to meet the energy needs for regenerating the solvent in all simulated scenarios. However, compression and liquefaction consumes more power than CC process itself
Stec et al. [26]	Reducing the energy efficiency design index for ships through a post-combustion CC process	47,000 DWT, 9960 kW HFO is used	Medium range tanker	MEA	Utilizing waste heat in post-combustion CC is a viable means to meet Energy Efficiency Design Index (EEDI) requirements	A simulation study examining the CC process is conducted under diverse ambient conditions such as arctic, ISO, and tropical environments. The capture rate is adjusted to match the available waste heat, and exhaust gas desulphurization is implemented to safeguard the amine solvent from degradation
Ji et al. [141]	Post-combustion CC for tank to propeller via process modelling and simulation	11,700 kW dual-fuel MFO is used	Tanker with dual-fuel	MEA, DIPA, and MDEA-PZ	A blended-amine maritime CC model is firstly proposed with good validation outputs, and MDEA-PZ system could capture more than 57% CO_2_ and save 25% specific reboiler duty compared to MEA system	40 simulations are conducted to identify the optimal combination of solvents, absorber/stripper packing, and liquid-to-gas ratio in the CC process
Güler and Engin [121]	An investigation on the solvent-based CC and storage system by process modelling and comparisons with another carbon control methods for different ships	27.44 MW for VLCC, 2 × 16.02 MW for large conventional, 2 × 19.88 MW for Q-Flex and 2 × 22.72 MW for Q-Max @100% MCR	A VLCC tanker and three different sizes of LNG carriers (Q-Max, Q-Flex, and conventional LNG carrier)	35 wt% MEA	Compared different carbon control methods—speed reduction, LNG usage, and CCS. Speed reduction is found the most cost-effective for low-freight ships, while CCS is more cost-effective for high-speed, high-freight ships like Q-Max and Q-Flex and LNG carriers	CCS system cost analysis for ships showed equipment and capital costs had a greater impact on total life cycle costs than operating expenses. Capture equipment comprised 21.6%, and liquefaction equipment 27.2% of total life cycle costs for VLCC tankers. Moreover, as CO_2_ emissions increased, the total life cycle cost per captured CO_2_ decreased
Ros et al. [86]	Advancements in ship-based CC technology on-board of LNG-fuelled ships	LNG-fuelled ships	Not specified	30 wt% MEA, 30–40 wt% PZ, 2–28 wt% aqueous ammonia	MEA is found to be the solvent of choice for OCCS technology. Proper heat integration in the OCCS technology on LNG-fuelled ships can drastically reduce the OPEX of the process, making the costs CAPEX dependent	The cost of CO_2_ capture for the OCCS process on-board of the Sleipnir ship is calculated at 119 €/ton CO_2_ at an effective capture rate of 72.5%
Einbu et al. [120]	Energy assessments of onboard CO_2_ capture from ship engines by MEA-based post-combustion capture system with flue gas heat integration	LNG or Diesel	Not specified	30 wt% MEA	Ship engine exhaust alone is insufficient to meet the thermal energy needs of a CO_2_ capture unit operating above 50%. A fuel afterburner is necessary, leading to a 6–9% increase in fuel consumption with LNG and an 8–12% increase with diesel as the fuel source	In MEA-based CO_2_ capture, absorber height has a limited effect on energy consumption, especially at lower capture rates. Nevertheless, it remains crucial for optimizing the trade-offs between capital and operating expenses (CAPEX/OPEX)
Kawamata et al. [35]	Development of onboard CO_2_ capture system	88,715 DWT, 9960 kW	Coal carrier operated by ‘K’ Line	Not specified	N/A	Outcome of project ‘CC-Ocean’. World’s first demonstration project of a marine-based CO_2_ capture system on actual voyage to successfully separate and capture CO_2_ from flue gas
MMMCZCS [94]	The role of onboard CC in maritime decarbonization	LSFO, LNG, Methanol	Container (15,000 TEU), Tanker-LR2, Tanker-VLCC (300,000 DWT), Bulk-Kmax (82,000 DWT), Bulk-Capesize (205,000 DWT)	Amine based	Newbuilds are more promising for OCC, as retrofits are costly and can require major modifications. Partial CC has higher CO_2_ abatement costs due to high initial CAPEX. Large tankers offer the best business cases, while small bulk carriers face more challenges	The results from a work package on onboard CC completed as part of the Green Fuels Optionality Project (GFOP)
Negri et al. [89]	Navigating within the safe operating space with CC on-board	8500 TEU ICE engine with HFO	Container ship	30 wt% MEA	The capture on-board scenario can achieve 94% efficiency on the net CO_2_ emissions at 85 $/tCO_2_ while substantially reducing impacts on core planetary boundaries	CCS outperforms direct air capture (DAC), decreasing the carbon footprint of the set scenario by 52%
Negri et al. [90]	Sustainable Development Goals assessment of CC on-board	A standard internal combustion engine with HFO	8500 TEU container ship	30 wt% MEA	Upgraded container ship with CC reduces emissions by 94% at $85 per ton of CO_2_. Overall, it diminishes climate change impact by 50%, while the standalone direct air capture technology achieves a 45% reduction	The results are assessed in relation to five Sustainable Development Goals, utilizing 16 life cycle impact assessment metrics and their corresponding absolute thresholds
Wang et al. [125]	Study on optimization and characteristics of ship-based CC system based on intercooling process	3000 kW dual-fuel LNG is used	LNG ship	25–40%wt MEA	Intercooling process consistently captured more carbon than the basic one. With solvent flow ratio adjustment, intercooling showed a 6.60% increase in capture and a 12.27% reduction in solvent usage compared to the base process	The unit CC energy consumption magnified only 0.201 MJ/kgCO_2_
Dong et al. [123]	Ship-based CC of LNG ships under off-design conditions	8000 DWT, 3000 kW dual-fuel LNG is used	LNG cargo ship	MEA	The liquid-to-gas ratio for optimal CC efficiency in the ship's main engine is approximately linearly related to both design and off-design conditions, correlating with the main engine load, at 100% MCR, a 72.38% decarbonization rate is achieved at 6.118 GJ/T CO_2_, and at 50% MCR, nearly 93.24% decarbonization is achieved at 11.023 GJ/T CO_2_	At 75% MCR main engine load, treating 73.7–100% of exhaust gas (10,088–13,691 kg/h) with a recirculation flow of 10,120–11,080 kg/h achieves a decarbonization rate exceeding 49%
Bayramoğlu [91]	Application of post-combustion CC process in marine diesel engine	5400 kW @100% load	Not specified	25 wt% MEA	The optimal exhaust absorber inlet temperature is 50 °C, resulting in an average CC performance of 95%. In the applied system, the EEDI is reduced by 14% with the ORC system and by 90% with the CC system	The ORC system turbine output power varies between approximately 414 kW and 606 kW for different CC system inlet temperatures under 100% load conditions
Güler and Ergin [124]	An investigation of the cooling, heating and power (CHP) systems integration with CC and storage for LNG carriers	CLNG (31,400 kW), Q-Flex (39,300 kW), Q-Max (45,200 kW)	Q-Max (265,000 m3), Q-Flex (210,000 m3) and CLNG (150,000 m3) LNG-fuelled engines	30% PZ	Employing a CCS system is more cost-effective than paying the European Union carbon tax for LNG carriers	The ORC-based CHP system raises the total Life Cycle Cost (LCC) due to initial expenses and efficiency issues. However, CHP integration helps mitigate some of the exergy losses incurred by the CCS system
Hua et al. [66]	Research progress of CCS technology based on the shipping industry	Not specified	Not specified	N/A	Covers the research progress on OCCS including CO_2_ transportation	Challenges and risks for CO_2_ sequestration is discussed

**Table 23:** Reports/papers on post-combustion chemical absorption for the application onboard ships

Authors (year)	Aim of the study	Engine type	Ship type	Solvent	Key findings	Additional information
Erto et al. [96]	Utilization of alumina-supported K2CO3 as CO_2_-selective sorbent: A promising strategy to mitigate the carbon footprint of the maritime sector	4.35 MW marine engine fuelled with marine gas oil and with a commercial seawater scrubber for SO_2_ removal	RoPax ferry	K_2_CO_3_ supported onto alumina	The supported K_2_CO_3_ can be completely regenerated with steam at 120 °C, the regeneration of the adsorption column takes double time than adsorption	A 30% CO_2_ emission cut can be attained for the analysed ship case study
Sharma and Maréchal [97]	CO_2_ Capture from Internal Combustion Engine exhaust using Temperature Swing Adsorption	Not specified	Not specified	PPN-6-CH_2-_TETA	The proposed system design ensures energy self-sufficiency by converting waste heat into mechanical power	Energy and exergy analyses are conducted, comparing it with the installation of fuel cells, using a truck as an example. This technology can be employed in conjunction with any ICE

**Table 24:** Reports/papers on membrane separation for the application onboard ships

Authors (year)	Aim of the study	Engine type	Ship type	Membrane type	Key findings	Additional information
Oh et al. [102]	Process design of onboard membrane CC and liquefaction systems for LNG-fuelled ships	36,500 kW @75% load LNG is used	53,200 DWT 13,800 TEU Container ship	Not specified	At a CO_2_/N_2_ selectivity of 50, energy consumption is higher than amine-based systems but decreases with improved selectivity, equipment size significantly reduces with higher permeance values, showing potential efficiency gains	This study evaluates different membrane configurations and for CO_2_ capture
Damartzis et al. [103]	Solvents for membrane-based post-combustion co2 capture for potential application in the marine environment	Not specified	Not specified	Membrane contactors	No solvent category can fully meet all ship requirements, Common solvents like secondary amines exhibit good compatibility with most key performance indicators (KPIs)	Newer options like phase change solvents and ionic liquids have the potential to outperform established solvents if they reach a comparable technological maturity level

**Table 25:** Reports/papers on Cryogenic CC for the application onboard ships

Authors (year)	Aim of the study	Engine type	Ship type	Key findings	Additional information
Willson [108]	Evaluation of the Marine Application of Advanced CC Technology	Car carrier (12.614 kW), RoPax ferry (4.800 kW)	Car carrier (10.200 DWT), RoPax ferry (830 DWT)	The advanced cryogenic CC (A3C) process is both feasible and cost-competitive for onboard applications	The A3C process recovers cold energy to reduce energy demand. Case studies examine the effects of different fuels (LNG/MGO) and capture rates on ship design, stability, and economic considerations
decarbonICE [37]	decarbonICE: On-board ship technology	Not specified	Not specified	N/A	CO_2_ will be stored as dry ice onboard, and the concept includes an analysis of sediment storage for dry ice

**Table 26:** Reports/papers on liquid onboard storage of CO_2_

Authors (year)	Aim of the study	Suggested pressure and temperature	Criterion used	Key findings	Additional information
Seo et al. [112]	Comparison of CO_2_ liquefaction pressures for ship-based CCS chain	15 bar (− 27 °C)	Life cycle cost (LCC) including CAPEX and OPEX to determine optimal pressure	Liquefying CO_2_ at 6 bar (− 52.3 °C) is cost-effective for storage and transport but incurs high refrigeration energy costs	At 15 bar, 58% of costs are attributed to the liquefaction system, with operational costs outweighing capital costs 9–12 times
Bjerketvedt et al. [113]	Deploying a shipping infrastructure to enable CC and storage from Norwegian industries	15 bar (− 27 °C)	A net present value criterion is used to compare retrofitting of the 15-bar and 7-bar transport technology	Shipping at 7 bar is cheaper than 15 bar but faces challenges due to less mature technology at 7 bar	The 15-bar transport chain has an average cost of 32.4 €/ton, while the 7-bar chain costs 25.4 €/ton
